# Centrosome- and Golgi-Localized Protein Kinase N-Associated Protein Serves As a Docking Platform for Protein Kinase A Signaling and Microtubule Nucleation in Migrating T-Cells

**DOI:** 10.3389/fimmu.2018.00397

**Published:** 2018-03-01

**Authors:** Seow Theng Ong, Madhavi Latha Somaraju Chalasani, M. H. U. Turabe Fazil, Praseetha Prasannan, Atish Kizhakeyil, Graham D. Wright, Dermot Kelleher, Navin Kumar Verma

**Affiliations:** ^1^Lee Kong Chian School of Medicine, Nanyang Technological University, Singapore, Singapore; ^2^Institute of Medical Biology, A*STAR, Singapore, Singapore; ^3^Department of Medicine, University of British Columbia, Vancouver, BC, Canada; ^4^Department of Biochemistry and Molecular Biology, University of British Columbia, Vancouver, BC, Canada; ^5^Singapore Eye Research Institute, Singapore, Singapore

**Keywords:** T-cell migration, AKAP450, AKAP350, adaptor protein, centrosomal proteins, microtubules

## Abstract

Centrosome- and Golgi-localized protein kinase N-associated protein (CG-NAP), also known as AKAP450, is a cytosolic scaffolding protein involved in the targeted positioning of multiple signaling molecules, which are critical for cellular functioning. Here, we show that CG-NAP is predominantly expressed in human primary T-lymphocytes, localizes in close proximity (<0.2 μm) with centrosomal and Golgi structures and serves as a docking platform for Protein Kinase A (PKA). GapmeR-mediated knockdown of CG-NAP inhibits LFA-1-induced T-cell migration and impairs T-cell chemotaxis toward the chemokine SDF-1α. Depletion of CG-NAP dislocates PKARIIα, disrupts centrosomal and non-centrosomal microtubule nucleation, causes Golgi fragmentation, and impedes α-tubulin tyrosination and acetylation, which are important for microtubule dynamics and stability in migrating T-cells. Furthermore, we show that CG-NAP coordinates PKA-mediated phosphorylation of pericentrin and dynein in T-cells. Overall, our findings provide critical insights into the roles of CG-NAP in regulating cytoskeletal architecture and T-cell migration.

## Introduction

The migration of T-lymphocytes, i.e., their homing to lymphoid organs, recruitment to inflamed tissue sites and mounting an adaptive immune response against infection, is a complex, but precisely regulated physiological process. This requires coordinated signal transduction pathways that culminate in dynamic reorganization of the cytoskeletal systems and active T-cell locomotion. In contrast to other cell types, the migratory polarity and structural asymmetry of motile T-cells are unique in the way that the nucleus occupies the front region; whereas, the centrosome and Golgi complex remain close to the nucleus at the back. The generally accepted model, about motile T-lymphocytes, is that centrosome serves as a site for microtubule nucleation i.e., microtubule-organizing center (MTOC). The minus ends of microtubule networks are anchored at the centrosome. Radial arrays of highly dynamic microtubule plus ends extend from the centrosome toward the cell periphery that ensures regulated signal transduction and rapid cell migration. It has recently been noted that microtubule nucleation can also arise *via* centrosome-independent mechanisms from membranous organelles, contributing to the asymmetric microtubule networks in polarized cells (1).

The Centrosome- and Golgi-localized protein kinase N-associated protein (CG-NAP) is a family of A-kinase anchoring proteins (AKAPs) that coordinate discrete signaling events by simultaneously interacting with multiple enzymes, such as kinases or phosphatases, and facilitating the phosphorylation of specific molecular substrates (2, 3). We have previously shown that CG-NAP/AKAP450 (also known as AKAP350 or AKAP9), is a critical integrating component of the integrin LFA-1-induced signaling complex in the human T-cell line HuT78 (4). CG-NAP, originally identified as a regulator of intracellular membrane trafficking and cell cycle progression, is a large coiled-coil protein of about 450 kDa that localizes predominantly to the centrosome (5–7). This adaptor protein was later found to be involved in microtubule nucleation in various cell types (8–10). CG-NAP interacts with a variety of protein kinases [protein kinase A (PKA), PKN, and PKC] and phosphatases (PP1 and PP2A) (6) in addition to phosphodiesterase 4D (11), calmodulin (12), casein kinase 1δ/ε (13), CIP4 (14), Ran (15), and cyclin E/Cdk2 (16); although the functional implications of these interactions are not fully uncovered. Existing literature on the studies with CG-NAP are mostly confined to non-immune cell types. However, the role of this adaptor protein in T-lymphocytes and the mechanism by which this protein regulates T-cell motility remains elusive.

Here, we provide a strong evidence that microtubule nucleation in motile T-cells occurs at both centrosomal and non-centrosomal regions. The adaptor protein CG-NAP serves as a docking platform for the microtubule nucleation at the centrosomal and non-centrosomal regions. Further, we show that CG-NAP facilitates PKA-mediated phosphorylation of pericentrin and dynein in T-cells. Our results thus provide a novel molecular mechanism by which CG-NAP mediates LFA-1 signaling and T-cell migration.

## Materials and Methods

### T-Lymphocytes and Culture

Human primary peripheral blood lymphocyte (PBL) T-cells and other immune cell subtypes were isolated from buffy coats obtained from the blood transfusion services at National University Hospital and Health Sciences Authority, Singapore using Lymphoprep™ (Axix Shield) density gradient centrifugation or using MACS kits (Miltenyi Biotec). Experiments were approved by Nanyang Technological University Institutional Review Board (IRB-2014-09-007). HuT78 T-cell line was obtained from the American Type Culture Collection. Cells were cultured in Gibco™ RPMI1640 medium supplemented with 2 mM l-glutamine, 1 mM sodium pyruvate, 10% fetal calf serum and antibiotics (penicillin and streptomycin) as described previously (17, 18).

### Antibodies and Reagents

Anti-CG-NAP and anti-GM130 mouse monoclonal antibodies were purchased from BD Biosciences. Rabbit polyclonal anti-tubulin-γ antibody was from Biolegend. Rabbit polyclonal anti-GM130 was from MBL International. Anti-dynein IC and GAPDH mouse monoclonal antibodies were from Merck Millipore. Anti-PKARIIα monoclonal and polyclonal antibodies were purchased from Santa Cruz Biotechnology. Rabbit polyclonal anti-pericentrin and anti-TGN46 antibodies were procured from Abcam. FITC conjugated anti-α-tubulin, rabbit polyclonal detyrosinated α-tubulin, and anti-human IgG (Fc specific) antibodies, nocodazole, poly-l-lysine (PLL), and DMSO were from Sigma-Aldrich. Phospho-PKA substrate (RRXS*/T*) rabbit monoclonal antibody, rabbit polyclonal anti-acetylated α-tubulin antibody, and forskolin were from Cell Signaling Technology. Secondary antibodies included anti-rabbit and anti-mouse Alexa Fluor 568, Alexa Fluor 488, and Alexa Fluor 633 (Molecular Probes). Rhodamine-phalloidin, Alexa Fluor 488 conjugated anti-α-tubulin, and Hoechst 33342 were from Life Technologies. Brefeldin-A was from Calbiochem. Recombinant human IL-2 and SDF-1α were from Peprotech. Human ICAM-1/CD54 protein was from Sino Biological. Dharmacon pre-designed ON-TARGET*plus* SMARTpool siRNA against targeting CG-NAP or PKARIIα were from GE Life Sciences.

### T-Cell Migration Assay

Our well-characterized migration-triggering model system, where T-cells are stimulated through the LFA-1 receptor *via* crosslinking with physiological ligand ICAM-1, was used for the study (17–19). Briefly, 6- or 96-well tissue culture plate or 18 mm coverslips, depending on the assay type, were coated with 5 µg/ml anti-Fc-specific goat anti-human IgG in sterile phosphate buffered saline (PBS, pH 7.2) for 2 h at 37°C or overnight at 4°C. Following incubation, wells were washed with sterile PBS, followed by coating with 1 µg/ml rICAM-1-Fc at 37°C for 2 h. The wells were washed twice with PBS before seeding the cells. Migration assays on rICAM-1 contained 5 mM MgCl_2_ and 1.5 mM EGTA in the cell culture medium to induce the high affinity form of the LFA-1 receptor on T-cells (20).

### GapmeR-Mediated Knockdown (KD) of CG-NAP in T-Cells

We have recently developed a novel technique of gene silencing in T-cells using cell-permeating antisense oligonucleotide molecules, called “GapmeR” (21). Briefly, human primary T-cells or HuT78 cells (1 × 10^6^ cells) were transfected with 500 nM non-targeting (control) or CG-NAP-targeting GapmeR for 48 h. After 48 h, cells were collected and used. This method resulted in >90% KD of CG-NAP expression with high specificity, as determined by nanoString nCounter gene expression analysis by comparing the expression levels of a set of 192 different genes (21).

### T-Cell Transwell Chemotaxis Assay

The ability of LFA-1-stimulated T-cells to transmigrate toward the chemokine was determined using impedance-based measurements by xCELLigence real time cell analyzer system (ACEA Biosciences). Briefly, the upper chambers of CIM-plate 16 wells (5 µm pore; ACEA Biosciences) were pre-coated with rICAM-1-Fc (1 µg/ml) at 4°C overnight and blocked with 5% (*w/v*) bovine serum albumin (BSA, Sigma-Aldrich) for 1 h at 37°C. SDF-1α (50 ng/ml) was added into the lower chamber of selected wells as a chemoattractant. Serum starved T-cells were loaded in triplicate in the upper chambers and allowed to migrate toward 50 ng/ml SDF-1α-enriched medium in the lower wells at 37°C. Transwell T-cell migration was monitored continuously in real time over a 6-h period, automatically plotted in graph format and presented.

### Microtubule Regrowth Assay

Control or CG-NAP-depleted T-cells were seeded on rICAM-1- or PLL-coated coverslips and incubated at 37°C for 2 h as described above. Microtubules were depolymerized by incubating cells at 4°C or 10 µM nocodazole in RPMI for additional 40 min. Cells were washed with RPMI and incubated at 37°C for various time points [0 (control), 10, 20, and 60 s], depending on the experiments to allow microtubule regrowth. Cells were then fixed after recovery in 4% (v/v) formaldehyde (Sigma-Aldrich) and processed for immunofluorescence microscopy.

### Confocal and Super-Resolution 3D-Structured Illumination (3D-SIM) Microscopy

For confocal or 3D-SIM imaging and analysis, cells were seeded to rest or migrate on coverslips as described above and then fixed with 4% (v/v) formaldehyde (Sigma-Aldrich) for 10 min, followed by permeabilization using 0.3% Triton X-100 (Bio-Rad) in PBS for 5 min. Cells were blocked with 3% BSA (Sigma-Aldrich) for 30 min at room temperature and then labeled with appropriate primary and secondary antibodies for 60 and 30 min, respectively at room temperature. Samples were washed three times with PBS-0.1% Tween 20 (Bio-Rad) after each antibody labeling step. Hoechst 33342 was used to visualize the nucleus. After washing, coverslips containing immunostained cells were mounted on clear glass slides using Fluoromount™ Aqueous Mounting Medium (Sigma-Aldrich) for confocal microscopy or VECTASHIELD^®^ Mounting Medium (H-1000; Vector Laboratories) for 3D-SIM.

Confocal microscopy was performed using the LSM 710/800 Airyscan microscope equipped with 405, 488, and 561 nm lasers for excitation and bandpass emission filters and a Plan Neofluar 63× oil objective/1.4 numerical aperture (NA) oil immersion objective lens (Carl Zeiss, Inc.) The confocal pinhole was set to one Airy unit for the green channel and other channels adjusted to the same optical slice thickness. Figure preparation and image analysis were carried out using ZEN lite 2.1 (Carl Zeiss) and Imaris software (Andor-Bitplane, Zurich). 3D-SIM was performed using a DeltaVision OMX v4 Blaze microscope (GE Healthcare, Issaquah, WA, USA) equipped with 488 and 568 nm lasers, a solid-state illuminator (WF) for excitation, and BGR filter drawer (emission wavelengths 436/31 for 4,6-diamidino-2-phenylindole, 528/48 for Alexa 488, 609/37 for Alexa 568, and 683/40 for Alexa 642). An Olympus Plan Apochromat ×100/1.4 point spread function oil immersion objective lens was used with liquid-cooled Photometrics Evolve EM-CCD cameras (Photometrics, Tucson, AZ, USA) for each channel. Fifteen images per section per channel were acquired (made up of three rotations and five phase movements of the diffraction grating) at a *z*-spacing of 0.125 µm (22, 23). Structured illumination reconstruction or deconvolution followed by alignment was conducted using the SoftWorX (Applied Precision) programme with figure and video preparation in Fiji (24) and Imaris software.

### Microscopic Image Analysis

3D reconstructions of Z-stack confocal microscopic images were generated and quantified using Imaris software. To analyze Golgi fragmentation, we performed 3D volume surface rendering on Golgi staining as described previously (25). Distinct Golgi structures with object volume below <0.2 µm^3^ threshold were considered fragmented. In 3D volume-rendered images, the distance of PKARIIα from the centrosomal marker proteins was analyzed using ImarisCell™ module and PKARIIα spots that were within <0.2 µm to the centrosome were considered as confined to the centrosomal region. The FilamentTracer™ tool was used to trace microtubule filaments and quantify the length and total number of microtubules within an individual cell. The intensity profiling of specific proteins in fluorescently labeled confocal images was performed using the ZEN lite software (Carl Zeiss, Inc.).

### Co-Immunoprecipitation Protein–Protein Interaction Assay

T-cells were induced to migrate on rICAM-coated 6-well plate (4.5 × 10^6^ cells/sample) and then lysed in cell lysis buffer at 4°C for 30 min as described previously (26). Cellular lysates were centrifuged at 10,000 *g* for 10 min at 4°C and the supernatant fraction was used for immunoprecipitation overnight using 2 µg of the required antibody or normal IgG as a control antibody. Protein A/G plus agarose beads (25 µl/sample) were added to the cell lysates for another 2 h at 4°C. The beads containing immune complexes were washed five times using the wash buffer (20 mM HEPES pH 7.4, 0.1% Triton X-100, 130 mM NaCl, 10% glycerol, 1 mM PMSF, 10 mM sodium fluoride, 2 mM sodium vanadate, and protease inhibitor cocktail) and then boiled in sample buffer for 5 min. The immune-complex samples were resolved on SDS-PAGE and transferred to a nitrocellulose membrane for Western immunoblot analysis.

### SDS-PAGE and Western Immunoblotting

Following various treatment conditions, cellular lysates were prepared as above. The protein content of the cell lysates was determined by the Bio-Rad Protein Assay. Equal amount of cell lysates were resolved on either 4–10% gradient gel or 10% SDS-PAGE gel and subsequently transferred to nitrocellulose membranes. Membranes were blocked using 5% blotto in TBS-0.05% Tween 20 buffer for 1 h at room temperature, and then they were incubated overnight in primary antibody with gentle rocking at 4°C. After three washes in TBS-0.05% Tween 20, the membranes were incubated with HRP-conjugated secondary antibody for 1 h at room temperature. Membranes were then washed thrice with TBS-0.05% Tween 20 buffer, developed using ECL solution, and exposed to light sensitive films. Densitometry quantitation of individual protein bands was performed using ImageJ software.

### High-Content Analysis (HCA)

A HCA protocol for T-cell migration analysis has been optimized and established in our laboratory (20, 27). Briefly, cells were seeded in triplicate in 96-well flat bottomed plates pre-coated with either PLL or rICAM-1 for 2 h. After washing, cells were fixed, permeabilized, and then stained with Rhodamine-Phalloidin, Alexa Fluor 488 conjugated anti-α-tubulin, and Hoechst. Plates were scanned (20× objective, nine randomly selected fields/well) using an automated HCA microscope IN Cell Analyzer 2200 (GE Healthcare). T-cell migratory phenotypes in the acquired images were automatically quantified by IN Cell Investigator software (version 1.6) using Morphology 1 analysis bio-application module (GE Healthcare).

### Statistical Analysis

Statistical significance of differences among two experimental groups was analyzed by two-tailed unpaired Student’s *t*-test. The level of significance was shown at three levels, *viz*., *0.05, **0.01, and ***0.001.

## Results

### CG-NAP Is Abundantly Expressed in Human Peripheral T-Cells

**Figure 1:**
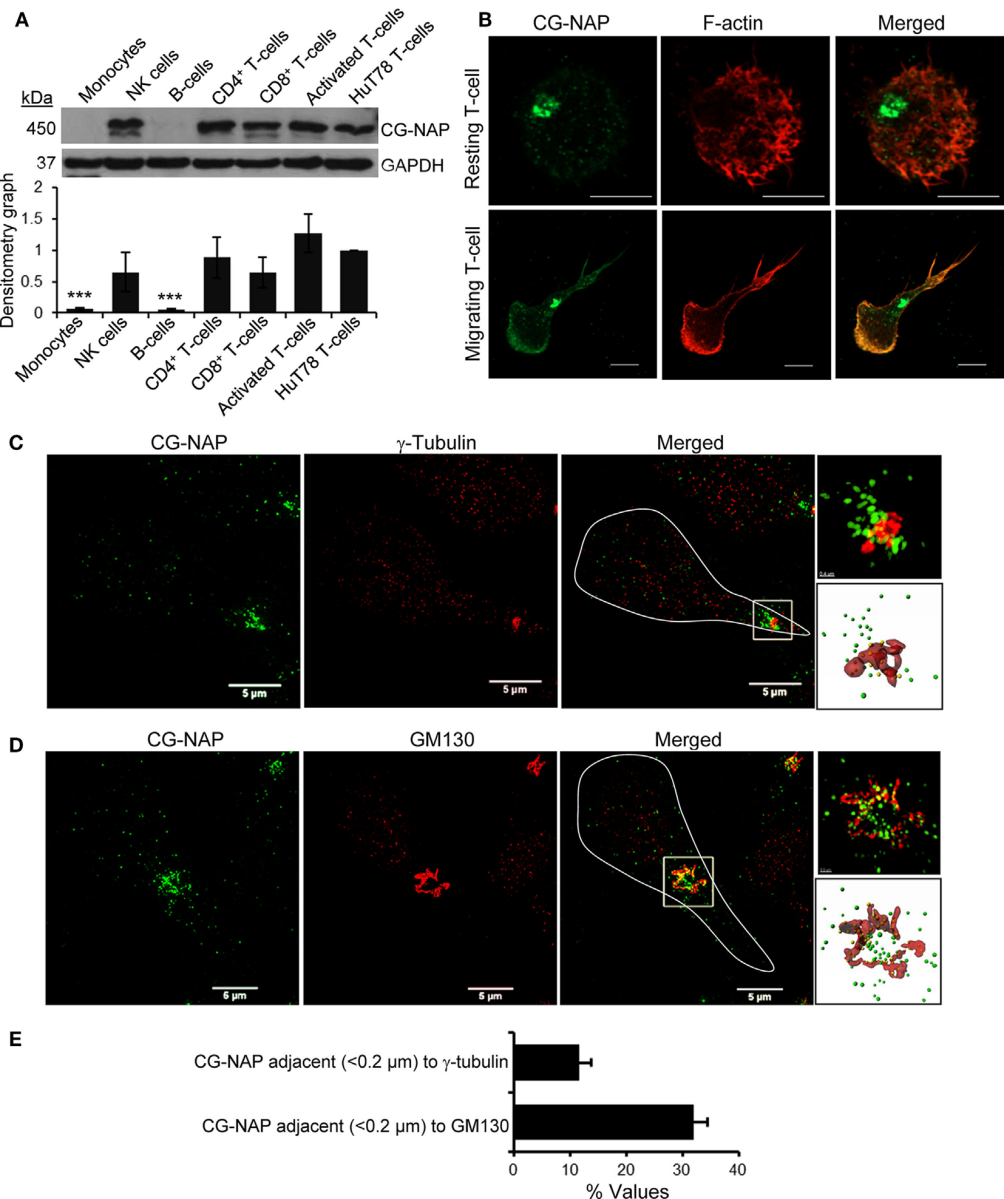
Centrosome- and Golgi-localized protein kinase N-associated protein (CG-NAP) expression and localization in human T-lymphocytes. **(A)** Cellular lysates from various immune cell subtypes, including monocytes, NK-cells, B cells, CD4^+^ T-cells, CD8^+^ T-cells, activated peripheral blood lymphocyte (PBL) T-cells, and HuT78 T-cell line were subjected to Western immunoblotting analysis for CG-NAP expression. GAPDH was used as a loading control. Relative densitometry graph of CG-NAP expression using immune cell subsets purified from four donors compared to HuT78 T-cells is presented (mean ± SEM, ***p < 0.001). Full-length blots are provided as Figure S9 in the Supplementary Material. **(B)** Control and LFA-1/ICAM-1-stimulated PBL T cells were immunostained with anti-CG-NAP (*green*) and Rhodamine-Phalloidin (*red*), and imaged by confocal microscopy. **(C,D)** LFA-1/ICAM-1-stimulated PBL T cells were immunostained with anti-CG-NAP (*green*) and anti-γ-tubulin [centrosome marker, *red*, **(C)**] or anti-GM130 [*cis*-Golgi marker, *red*, **(D)**], and analyzed by 3D-structured illumination. The motile T cell is white-outlined in merged panel. Zoomed-in images of the selected magnified region in the merged panels are shown for better visualization. The volume rendering of CG-NAP protein (*green* spots), which are in close proximity (<0.2 μm) to γ-tubulin or GM130 (*red* surfaces) are shown as yellow spots. **(E)** The fraction (*% values*) of CG-NAP protein spots adjacent (<0.2 μm) to γ-tubulin or GM130 is presented (mean ± SEM; *n* = 10 cells). Data represent at least three independent experiments. Scale bar, 5 µm.

Various subtypes of primary PBL T-cells express substantial amount of the 450 kDa protein CG-NAP (Figure 1A). Confocal microscopy shows that the majority of this adaptor protein is contained at the centrosomal region as distinct spots with some amount of the protein in the cytoplasm and on the membrane in both resting and LFA-1-stimulated PBL T-cells (Figure 1B). 3D-SIM imaging and subsequent volume-rendered reconstruction analysis reveal that about 12% of centrosomally located CG-NAP protein spots are present in close proximity (<0.2 µm) to γ-tubulin and about 31% CG-NAP spots within the region are adjacent (<0.2 µm) to the *cis*-Golgi protein GM130 in motile PBL T-cells (Figures 1C–E; Videos S1 and S2 in Supplementary Material).

### CG-NAP Plays a Crucial Role in T-Cell Migration and Chemotaxis

**Figure 2:**
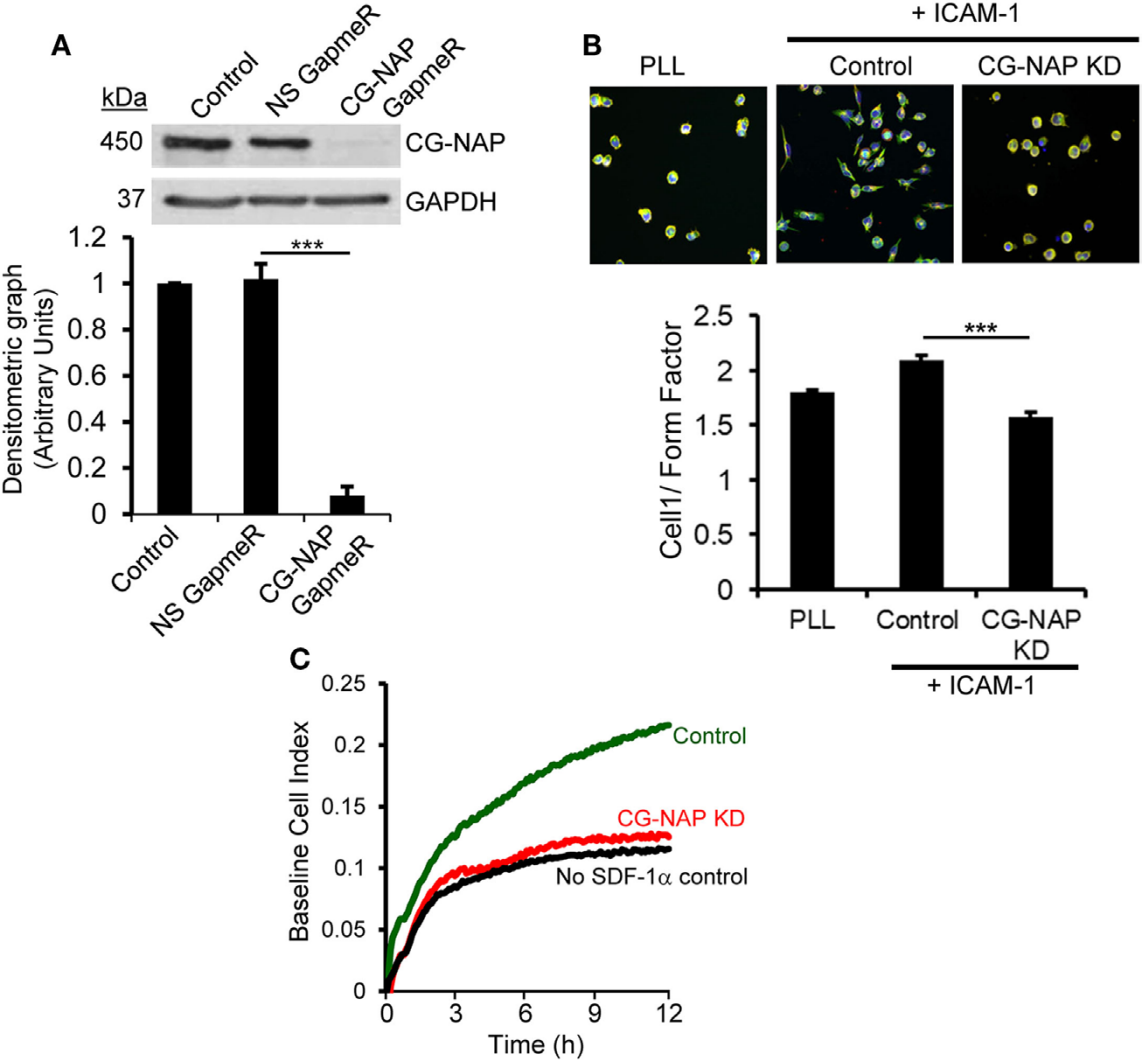
Centrosome- and Golgi-localized protein kinase N-associated protein (CG-NAP) expression is important for T-cell migration. **(A)** HuT78 cells (untreated *control*) were treated with non-specific GapmeR or CG-NAP-targeting GapmeR for 48 h and the expression of CG-NAP was analyzed by Western immunoblotting. Densitometric analysis of the blots for CG-NAP vs GAPDH is presented (*n* = 4 independent experiments). Full-length blots are provided as Figure S10 in the Supplementary Material. **(B)** Under similar conditions of knockdown (KD), HuT78 cells were seeded on poly-l-lysine (PLL, resting control) or on rICAM-1-coated 96-well plates. Cells were fluorescently stained with Rhodamine-Phalloidin (*red*), Alexa Fluor 488 conjugated anti-α-tubulin (*green*), and Hoechst 33342 (*blue*), imaged using an IN Cell Analyzer 2200 automated microscope and T-cell migratory phenotypes (cell 1/Form Factor, obtained from >2,000 cells/well seeded in triplicates in three independent experiments) were automatically quantified by IN Cell Investigator software. **(C)** Migratory potential of control and CG-NAP KD peripheral blood lymphocyte (PBL) T-cells toward the chemokine SDF-1α was examined in real time by transwell migration assay using xCELLigence monitoring system. Data are representative of at least three independent experiments, bar diagram is mean ± SEM, ***p < 0.001.

To determine whether CG-NAP is essential for T-cell migration, we knocked down the expression of this protein in HuT78 T-cells (>90% KD) using our recently reported GapmeR-mediated gene silencing technique (21) (Figure 2A). Depletion of CG-NAP did not interfere with T-cell viability (data not shown). HuT78 T-cells incubated on immobilized rICAM-1 displayed typical T-cell migratory phenotypes with distinct lamellipodia at the leading edge and uropod at the trailing end (Figure 2B). However, the LFA-1/ICAM-1-induced migratory phenotypes were significantly impaired in CG-NAP-depleted HuT78 T-cells. CG-NAP-depleted T-cells remained rounded on rICAM-1-coated surfaces similar to resting cells on PLL-coated surfaces, indicating the inability of CG-NAP KD T-cells to migrate. Quantification of the migratory phenotypes by HCA algorithm (27) showed significant decrease in cell 1/form factor from 2.098 in control cells to 1.58 in CG-NAP-depleted T-cells (Figure 2B). Furthermore, we observed significantly reduced transwell migration of CG-NAP-depleted PBL T-cells compared to control PBL T-cells through rICAM-1-coated membrane toward the chemokine SDF1α (Figure 2C).

### CG-NAP Is a Centrosomal Docking Platform for PKA and Pericentriolar Material (PCM) in T-Cells

**Figure 3:**
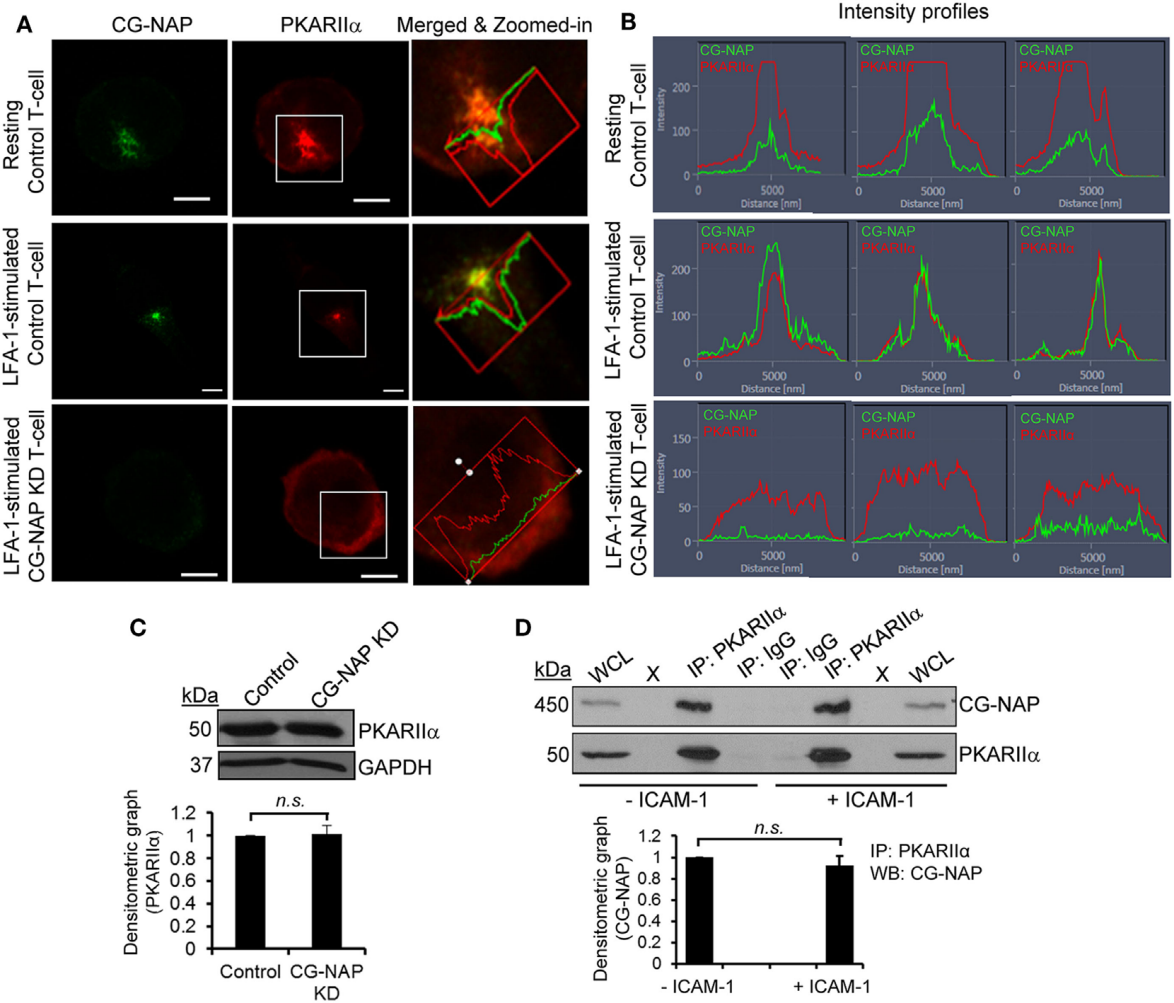
Centrosome- and Golgi-localized protein kinase N-associated protein (CG-NAP) regulates protein kinase A localization in T-lymphocytes. **(A)** Control and CG-NAP knockdown (KD) HuT78 T-cells were either unstimulated (*resting T-cell*) or stimulated *via* LFA-1/ICAM-1 for 1 h (*LFA-1-stimulated T-cell*) and fixed. Cells were immunostained with antibodies against CG-NAP (*green*) and PKARIIα (*red*) and analyzed by confocal microscopy. To better visualize the proximity of both proteins, merged and zoomed-in images with protein intensity profiles are shown. Scale bar: 5 µm. **(B)** Fluorescence signal intensity profiles of CG-NAP and PKARIIα proteins in confocal images obtained from three different sets of experiments are shown. **(C)** Western blots and relative densitometric graph of PKARIIα vs GAPDH in control and CG-NAP KD lysates (mean ± SEM). **(D)** Cellular lysates from unstimulated or rICAM-1-stimulated HuT78 cells were immunoprecipitated using either anti-PKARIIα antibody or control IgG antibody. Immunoprecipitates were resolved on SDS-PAGE and subjected to Western blotting with anti-CG-NAP and anti-PKARIIα antibodies. Whole cell lysates (*WCL*, 10 µg each) were used as input controls for Western immunoblots; gel lanes indicated by “X” are empty lanes, i.e., no protein loaded. Full-length blots are provided as Figure S11 in the Supplementary Material. Data represent at least three independent experiments; *n.s*., non-significant.

We next hypothesized a crucial role for CG-NAP in co-ordinating the localization and enzyme activity of PKA toward its substrates at centrosome and Golgi apparatus. Indeed, we detected a close adjacency of CG-NAP with the regulatory subunit of protein kinase A (PKARIIα), predominantly at the centrosomal region in both resting and migrating T-cells (Figure 3A; upper and middle panels). The signal intensity profiles of both proteins in confocal images and 3D-SIM projection showed that most of the endogenous CG-NAP coexists with PKARIIα in T-cells (Figures 3A,B; Video S3 in Supplementary Material). Further quantification of multiple confocal images by volume rendering showed that about 15–40% of centrosomally located CG-NAP spots were in close proximity (<0.2 μm) to PKARIIα in resting and LFA-1-stimulated T-cells. KD of CG-NAP in T-cells caused loss of PKARIIα centrosomal localization (Figures 3A,B; lower panels). Of note, total protein levels of PKARIIα remained unaffected on CG-NAP KD as shown by Western immunoblotting (Figure 3C). Co-immunoprecipitation experiments further confirmed a direct interaction between CG-NAP and PKARIIα proteins in both resting and migrating HuT78 T-cells (Figure 3D) suggesting that CG-NAP strongly anchors PKA at the centrosomal region. It also suggests that the interaction between CG-NAP and PKA is not influenced by T-cell polarization on rICAM-1.

**Figure 4:**
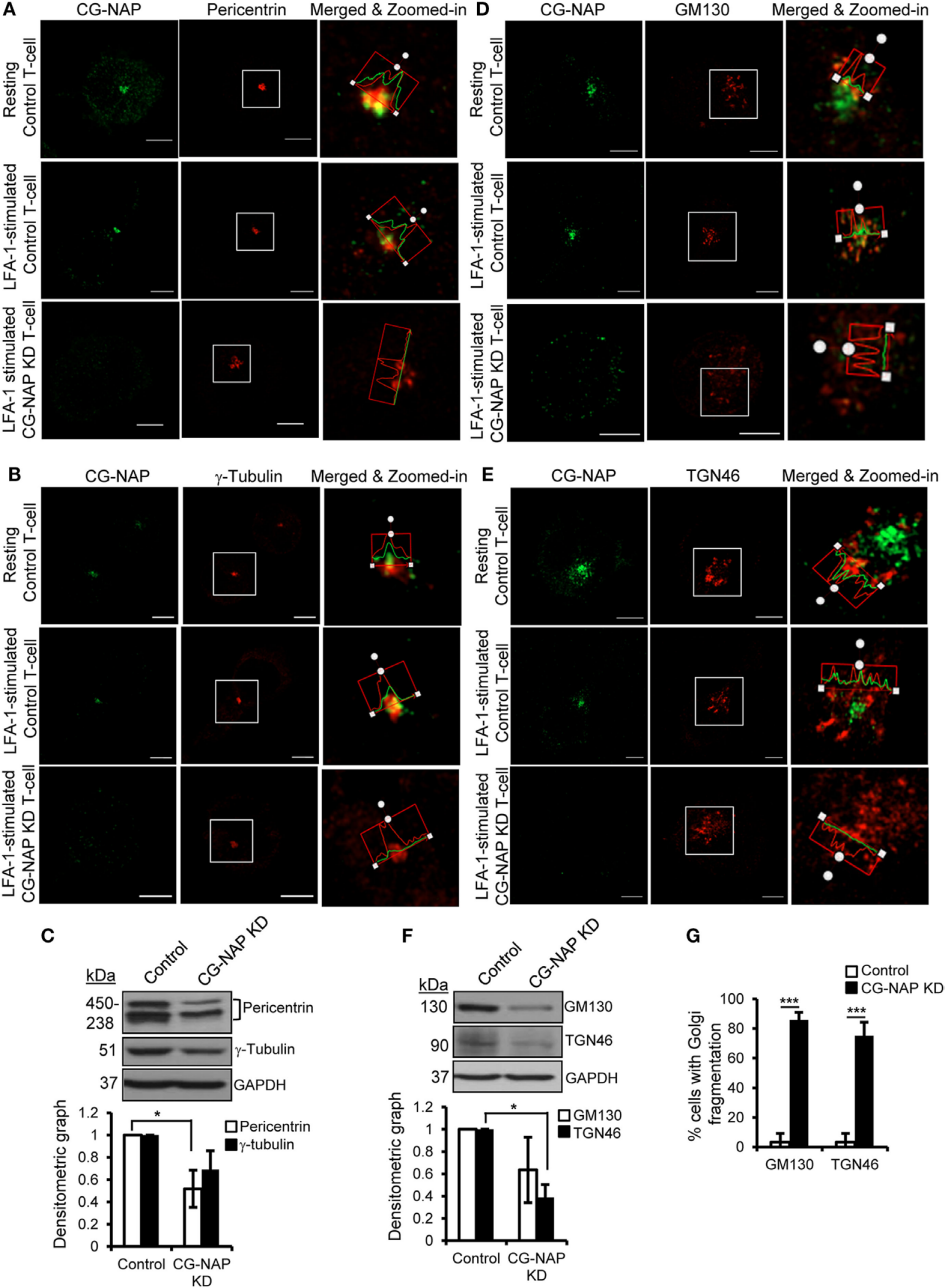
Centrosome- and Golgi-localized protein kinase N-associated protein (CG-NAP) is required for the confinement of centrosomal proteins and maintenance of Golgi architecture in T-lymphocytes. Control and CG-NAP knockdown (KD) HuT78 T cells were either unstimulated (*resting T-cell*) or stimulated *via* LFA-1/ICAM-1 for 1 h (*LFA-1-stimulated T-cell*) and fixed. Cells were immunostained with antibodies against CG-NAP (*green*) and pericentrin [**(A)**, *red*], γ-tubulin [**(B)**, *red*], GM130 [**(D)**, *red*], or TGN46 [**(E)**, *red*] and analyzed by confocal microscopy. Images of at least 10 different cells in three independent experiments were captured and representative sets of images are shown. Note: the intensity of pericentriolar material (PCM)/Golgi proteins in the confocal images should be considered as qualitative, but not quantitative representation of protein expression. To better visualize protein adjacency, merged and zoomed-in images with protein intensity profiles are shown. Scale bar: 5 µm. Western blots and densitometry graphs (mean ± SEM) showing expression levels of pericentrin, γ-tubulin **(C)**, GM130, and TGN46 proteins **(F)** in control and CG-NAP *KD* cells. The pericentrin blot shows multiple bands as also reported earlier (28, 29), which could be due to its numerous alternative splice forms (30). Full-length blots are provided as Figure S12 in the Supplementary Material. **(G)** Bar graph showing percentage of cells with Golgi breakdown in control and CG-NAP KD HuT78 T cells (mean ± SEM of 30 cells), as quantified by Imaris object rendering software. Golgi structures with object volume <0.2 μm^3^ were considered fragmented. As an example, a set of representative volume-rendered images is provided as Figure S13 in the Supplementary Material. Data represent at least three independent experiments, *p < 0.05; ***p < 0.001.

**Figure 5:**
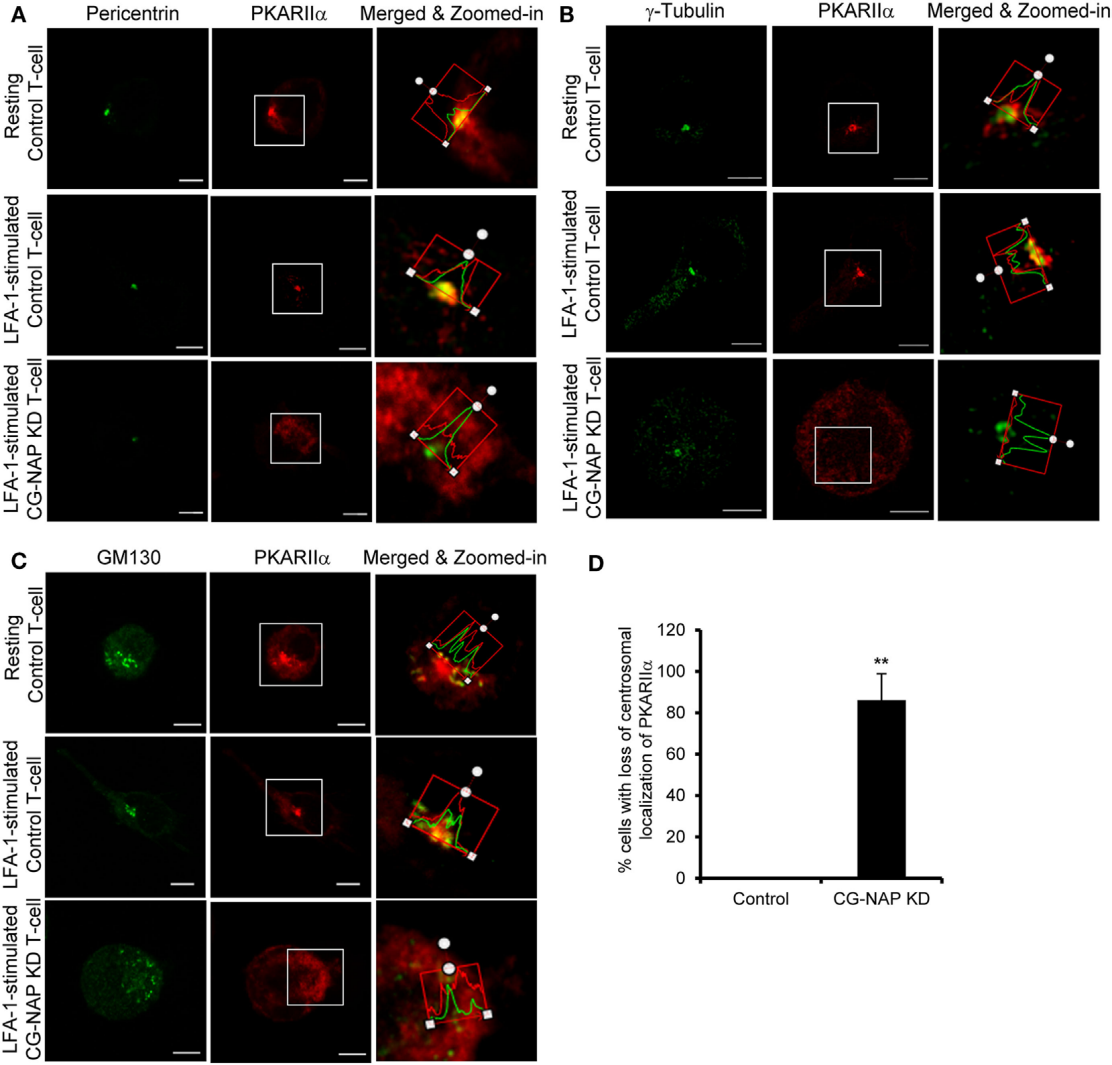
Centrosome- and Golgi-localized protein kinase N-associated protein (CG-NAP) regulates PKARIIα interaction with pericentriolar material and Golgi proteins. Control and CG-NAP knockdown (*KD*) HuT78 T cells were either unstimulated (*resting T-cell*) or stimulated *via* LFA-1/ICAM-1 for 1 h (*LFA-1-stimulated T-cell*) and fixed. Cells were co-immunostained with antibodies against PKARIIα (*red*) and pericentrin [**(A)**, *green*], γ-tubulin [**(B)**, *green*], or GM130 [**(C)**, *green*] and analyzed by confocal microscopy. Images of at least 10 different cells in three independent experiments were captured and representative sets of images are presented. To better visualize the proximity of proteins, merged and zoomed-in images with protein intensity profiles are shown. Scale bar: 5 µm. **(D)** Bar graph showing percentage of cells with loss of centrosomal localization of PKARIIα in control and CG-NAP KD cells (mean ± SEM of >65 cells, **p < 0.01), as quantified by ImarisCell module software. PKARIIα spots <0.2 μm distance to centrosome were considered as confined to the centrosomal region. As an example, a set of representative volume-rendered images is provided as Figure S14 in the Supplementary Material. Data represent three independent experiments.

To further investigate if CG-NAP regulates the centrosome and Golgi structures, control or CG-NAP KD HuT78 T-cells (resting and LFA-1-stimulated) were co-immunostained for CG-NAP and pericentrin or γ-tubulin (centrosomal marker proteins) or *cis*-Golgi marker GM130 or *trans*-Golgi marker protein TGN46. Confocal microscopy and subsequent analysis using protein signal intensity profiling showed that CG-NAP was present in adjacency with PCM and Golgi proteins in both resting as well as in LFA-1-stimulated migrating HuT78 T-cells (Figures 4A,B,D,E upper and middle panels; intensity profiles of multiple confocal images are provided as Figure S1 in Supplementary Material). Further volume-rendering analysis of confocal images showed that a fraction of CG-NAP spots was in close proximity (<0.2 μm) to pericentrin (10–14%), γ-tubulin (13–20%), GM130 (24–28%), and TGN46 (12–13%) in resting and LFA-1-stimulated HuT78 T-cells. While both pericentrin and γ-tubulin proteins remained intact at the centrosome in CG-NAP-depleted HuT78 T-cells (Figures 4A,B, lower panels), their expression levels were reduced in CG-NAP-depleted cells in comparison to control cells (Figure 4C). Substantial decrease in GM130 and TGN46 protein levels were also observed in CG-NAP-depleted HuT78 T-cells (Figure 4F). Similar decrease in the expression of GM130, TGN46, and pericentrin was recorded with siRNA-induced CG-NAP KD in both primary PBL and HuT78 T-cells (Figure S2 in Supplementary Material), suggesting the possibility of proteolytic degradation of these proteins in the absence of CG-NAP. Notably, CG-NAP KD caused breakdown of *cis*- and *trans*-Golgi structures in LFA-1-stimulated HuT78 cells (Figures 4D,E, lower panels). Depletion of CG-NAP caused partial to complete breakdown of GM130 in 85.8 ± 5.2% T-cells and TGN46 disturbance in 75.2 ± 9.07% cells (Figure 4G). Similar disruption of Golgi architecture and inhibition of cell migration were observed in HuT78 cells pre-treated with nocodazole or brefeldin A, used as controls (Figure S3 in Supplementary Material). Additionally, we observed close proximity (<0.2 μm) of substantial amount of pericentrin (88–92%), γ-tubulin (65–73%), and GM130 (8–13%) spots with PKARIIα in both resting and LFA-1-stimulated control T-cells (Figures 5A–C upper and middle panels; intensity profiles of multiple confocal images are provided as Figure S4 in Supplementary Material). Depletion of CG-NAP expression profoundly disturbed the confinement of these PCM proteins and caused Golgi fragmentation (Figures 5A–C lower panels). An average of 86.1 ± 12.7% CG-NAP KD cells and none of control cells showed absence of centrosome localization of PKARIIα (Figure 5D). These results suggest that CG-NAP plays a crucial role in the confinement of PCM protein complexes and docking of PKA to the centrosome in human T-cells.

### CG-NAP Is Essential for the Localization of PKARIIα and PCM Proteins at the Microtubule Nucleation Sites in Motile T-Cells

**Figure 6:**
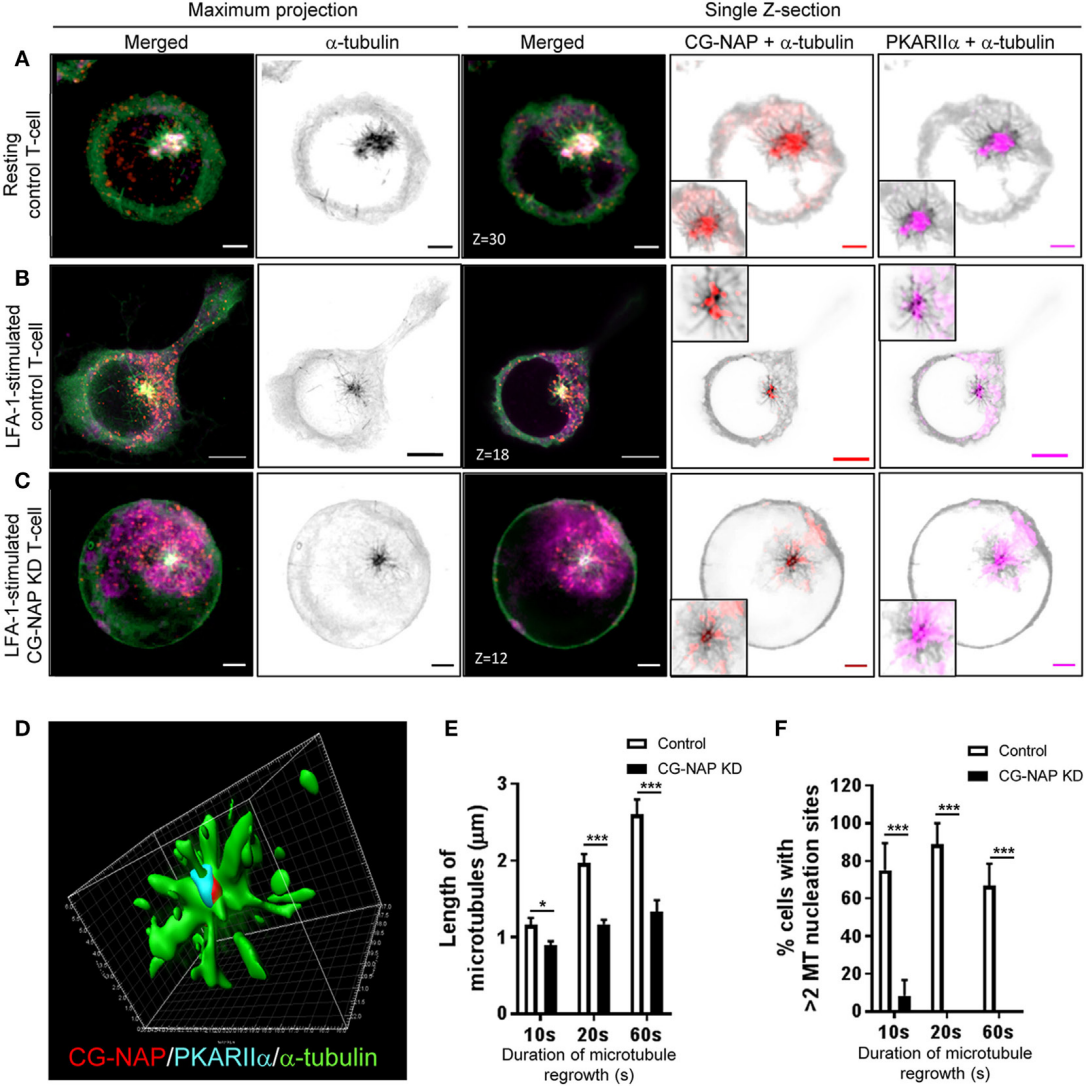
Centrosome- and Golgi-localized protein kinase N-associated protein (CG-NAP) regulates PKARIIα at the centrosomal microtubule nucleation in motile T-cells. Control and CG-NAP knockdown (*KD*) HuT78 T-cells were either unstimulated (*resting T-cell*) or stimulated *via* LFA-1/ICAM-1 for 2 h (*LFA-1-stimulated T-cell*), subjected to microtubule regrowth assay for 10 s and fixed. Cells were co- immunostained for CG-NAP (*red*), α-tubulin (*green*), and PKARIIα (*magenta*) and analyzed by confocal microscopy. Microtubule images were inverted into gray-scale by the Zen lite software and selected regions were magnified as insets for better visualization. Representative images of **(A)** control unstimulated, **(B)** LFA-1-stimulated, and **(C)** CG-NAP-depleted T cells are shown. Scale bar: 2 µm. **(D)** 3D-structured illumination projection of microtubule nucleation (α-tubulin, *green*) from CG-NAP (*red*) and PKARIIα (*cyan*) encapsulated complex using Imaris software. Images of at least 10 different cells in three independent experiments were captured and representative sets of images are presented. Images were automatically quantified using Imaris software and the average length of microtubules per cell **(E)** and percentage cells with >2 microtubule nucleation sites **(F)** in control and CG-NAP KD HuT78 T cells at 10, 20, or 60 s time-points are presented (mean ± SEM of >33 cells, *p < 0.01; ***p < 0.001).

**Figure 7:**
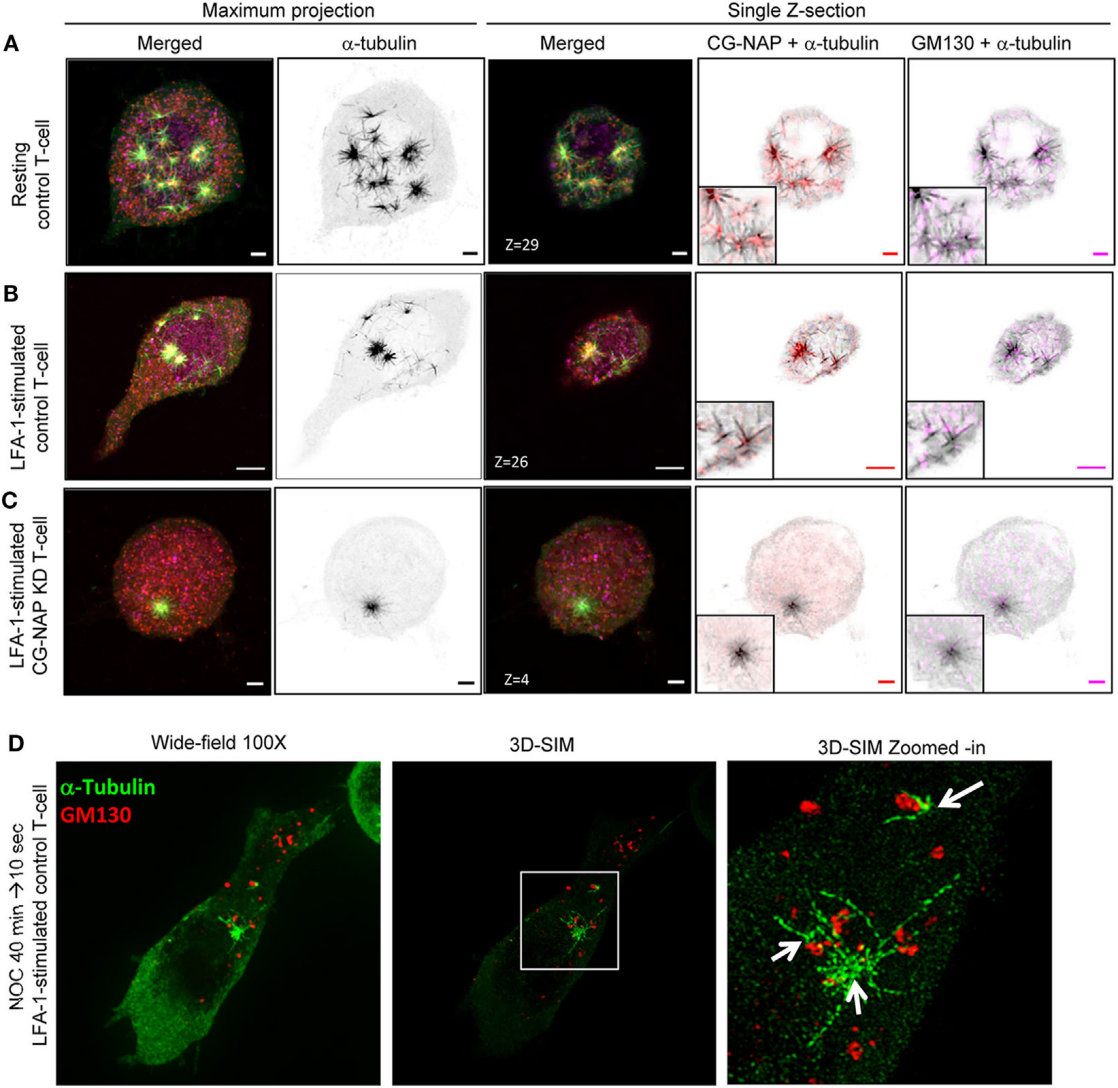
Non-centrosomal microtubule nucleation sites in T-lymphocytes. Control and centrosome- and Golgi-localized protein kinase N-associated protein (CG-NAP) knockdown (*KD*) HuT78 T-cells were either unstimulated (*resting T-cell*) or stimulated *via* LFA-1/ICAM-1 for 2 h (*LFA-1-stimulated T-cell*), subjected to microtubule regrowth assay for 10 s and fixed. Cells were co-immunostained for CG-NAP (*red*), α-tubulin (*green*), and GM130 (*magenta*) and analyzed by confocal microscopy. Microtubule images were inverted into gray-scale by the Zen lite software and selected regions were magnified as insets for better visualization. Representative images of **(A)** control unstimulated, **(B)** LFA-1-stimulated, and **(C)** CG-NAP-depleted T-cells are shown. Scale bar: 2 µm. **(D)** LFA-1-stimulated HuT78 T-cells were treated with 10 µM nocodazole for 40 min followed by 10 s recovery at 37°C and fixed. Cells were co-immunostained with anti-GM130 (*red*) and α-tubulin (*green*) antibodies, and analyzed by wide-field microscopy and 3D-structured illumination in parallel at 100×. Zoomed-in images are shown for better visualization. Images of at least 10 different cells in three independent experiments were captured and representative sets of images are presented.

T-cells are unique, when compared to other mammalian cell types, not only with respect to their size, but also because they reorient their centrosome and Golgi to the uropod behind the nucleus during migration. Owing to the cell-type-specific behavior of the primary and secondary MTOC (centrosome and Golgi) (31), we set out to investigate the role of CG-NAP in T-lymphocyte microtubule nucleation. For this purpose, we performed microtubule regrowth assay to examine the recovery pattern of microtubules in control and CG-NAP-depleted HuT78 T-cells following LFA-1 stimulation. Centrosomal microtubule asters originating from the protein complexes consisting of CG-NAP, PKARIIα, pericentrin, and γ-tubulin were seen immediately after the initiation of microtubule regrowth (10 s) in both resting and migrating control T-cells (Figures 6A,B and 7A,B; Figure S5 in Supplementary Material). These microtubule arrays further extended at 20 s leading to a complete microtubule network formation by the end of 60 s (Figure S6 in Supplementary Material). CG-NAP formed encapsulated complex with PKARIIα during microtubule nucleation (Figure 6D; Video S3 in Supplementary Material). However, in CG-NAP KD T-cells, microtubule networks failed to regrow properly as compared to control cells (Figures 6C and 7C; Figures S5C,F and S6 lower panels in Supplementary Material). Imaris-based image quantification showed that the length of newly polymerized microtubule fibers was significantly less in CG-NAP KD cells compared to the control cells following LFA-1 stimulation (Figure 6E). There was a significant decrease in the number of microtubule steric filaments per cell in CG-NAP depleted cells compared to control cells (Figure S7 in Supplementary Material).

Furthermore, the depolymerization of T-cell microtubules disassembled the centrosomal localization of CG-NAP and a substantial amount of this protein appeared as punctate structures dispersed throughout the cytoplasm in addition to the centrosome (Figures 6 and 7). Of note, we detected multiple microtubule nucleating sites (typically 3–5) in T-cells with re-growing microtubules (Figures 6A and 7A; Video S4 in Supplementary Material). The number of microtubule nucleation sites were restricted to one (or two in few cells) in CG-NAP KD cells (Figures 6C and 7C; Video S5 in Supplementary Material). Quantitative analysis of the microscopic images revealed that about 70% control T-cells had >2 microtubule nucleation origins, which was significantly reduced to less than 10% in CG-NAP-depleted cells (Figure 6F). Apart from the presence of CG-NAP at microtubule nucleation sites, we observed that PKARIIα, γ-tubulin, and pericentrin remained associated with CG-NAP at these sites in resting and LFA-1-stimulated control T-cells (Figure 6; Figure S5 in Supplementary Material). Similar observations were recorded in primary T-cells (data not shown) suggesting that this phenotype could be a unique phenomenon in T-lymphocytes.

Notably, apart from the centrosomal region, we observed CG-NAP localization at the majority of the non-centrosomal microtubule nucleation sites in both resting and LFA-1-stimulated T-cells (Figures 6 and 7). 3D-SIM imaging of T-cells following treatment with nocodazole (that causes microtubule depolymerization similar to cold treatment) clearly showed that GM130 was not located at the microtubule nucleation origin, but at the close proximity to it (Figure 7D), suggesting that Golgi might not be the non-centrosomal nucleation origin in T-cells. Overall, microtubule regrowth experiments suggest that both the centrosomal and non-centrosomal microtubule nucleation occurs in a CG-NAP-dependent manner.

### CG-NAP Is Vital for Microtubule Distribution and α-Tubulin Post-Translational Modifications

**Figure 8:**
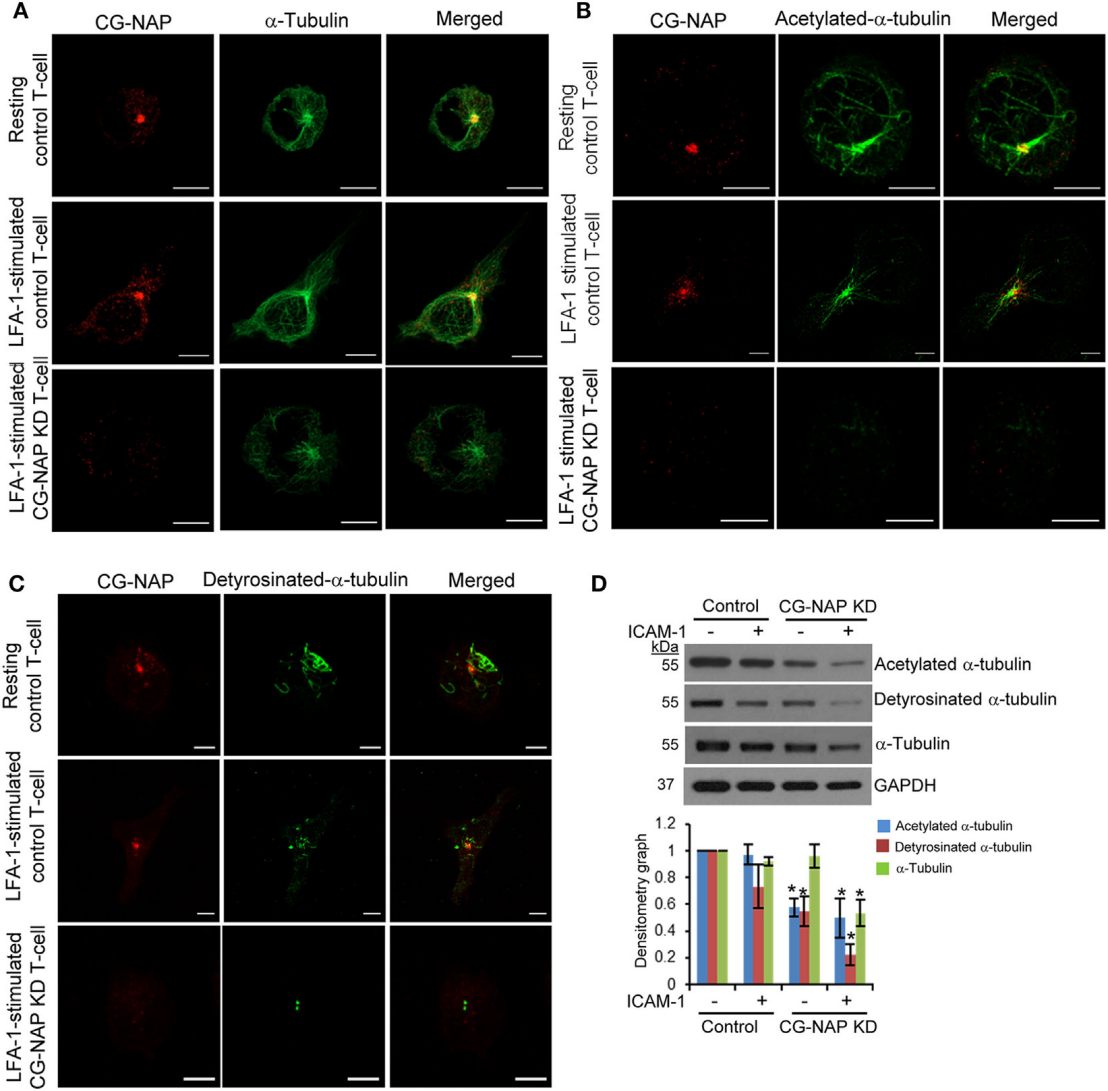
Centrosome- and Golgi-localized protein kinase N-associated protein (CG-NAP) knockdown (KD) in T-cells interferes with α-tubulin post-translational modifications. Control and CG-NAP *KD* HuT78 T cells were either unstimulated (*resting T-cell*) or stimulated *via* LFA-1/ICAM-1 for 1 h (*LFA-1-stimulated T-cell*) and fixed. Cells were co-immunostained with antibodies against CG-NAP (*red*) and α-tubulin [*green*, **(A)**], acetylated-α-tubulin [*green*, **(B)**] or detyrosinated-α-tubulin [*green*, **(C)**], and analyzed by confocal microscopy. Scale bar: 5 µm. Images of at least 10 different cells in three independent experiments were captured and representative sets of images are presented. **(D)** After cellular stimulation *via* LFA-1/ICAM-1 under similar experimental conditions, cells were lysed and post-translationally modified forms of α-tubulin were quantified by Western immunoblotting and densitometry analysis (mean ± SEM). Data represent three independent experiments, *p < 0.05. Full-length blots are provided as Figure S15 in the Supplementary Material.

Post-translational modifications of α-tubulin play a crucial role in α-tubulin-Golgi interaction, stability of microtubule networks, and positioning of Golgi and are functionally important for T-cell migration (32). LFA-1-induced migratory T-cells showed distinct distribution of microtubule arrays nucleating from the MTOC toward both leading edge and uropod (Figure 8A, upper and middle panels). In contrast, α-tubulin network appeared collapsed in CG-NAP depleted HuT78 cells (Figure 8A, lower panels). Acetylated α-tubulin networks were found to radiate from the centrosome in both resting and migrating control cells (Figure 8B, upper and middle panels), but were absent in CG-NAP-depleted HuT78 T-cells (Figure 8B, lower panels). Similarly, detyrosinated α-tubulin was contained as a compact MTOC structure in CG-NAP knockdown HuT78 cells unlike control cells that displayed intense staining emanating from the MTOC (Figure 8C, lower panels vs upper and middle panels). Quantification of the microtubule fibers showed significant reduction of the total length of acetylated as well as detyrosinated α-tubulin in CG-NAP-depleted T-cells (Figure S8 in Supplementary Material). Western immunoblot analysis further confirmed significantly reduced expression levels of both acetylated and detyrosinated forms α-tubulin in CG-NAP-depleted T-cells (Figure 8D).

### CG-NAP Coordinates PKA-Mediated Phosphorylation of Pericentrin and Dynein in T-Cells

**Figure 9:**
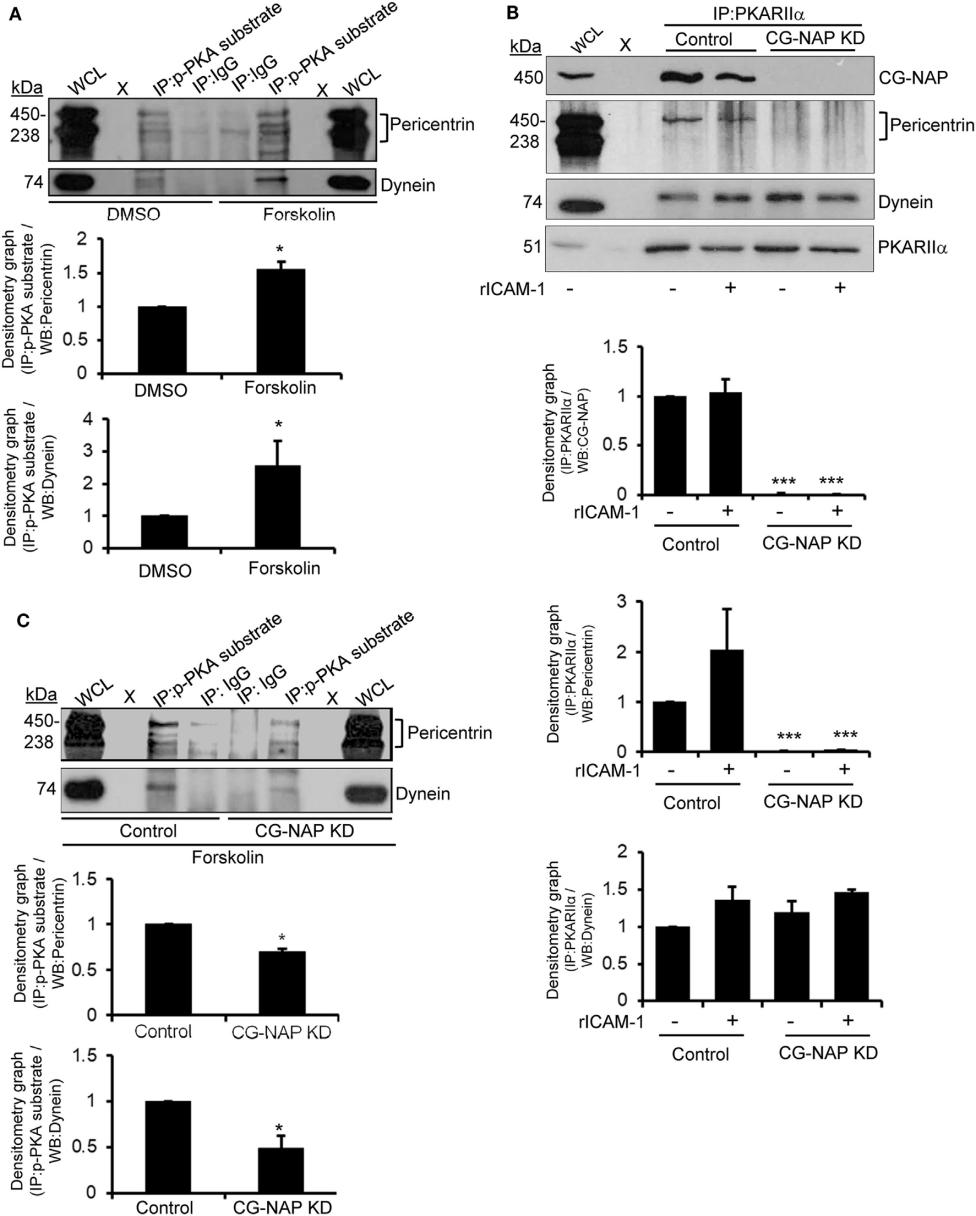
Immunoblot analysis of protein kinase A (PKA) substrates in T cells. **(A)** HuT78 cells were serum starved for 4 h and then treated with DMSO (*control*) or 30 µM forskolin for 30 min and lysed. Protein lysates were immunoprecipitated (IP) using either phospho-PKA substrate antibody (*p-PKA substrate*) or control IgG. Immunoprecipitates were resolved on SDS-PAGE and subjected to Western blotting with anti-pericentrin and anti-dynein antibodies. The pericentrin blot shows multiple bands as also reported earlier (28, 29), which could be due to its numerous alternative splice forms (30). **(B)** Control and CG-NAP knockdown (*KD*) HuT78 T-cells were either unstimulated or LFA-1-stimulated (+rICAM-1) for 1 h, lysed and IP using anti-PKARIIα antibody. Immunoprecipitates were resolved on SDS-PAGE and subjected to Western blotting with anti-CG-NAP, pericentrin, dynein, or PKARIIα antibodies. **(C)** Control or CG-NAP *KD* HuT78 cells were treated with 30 µM forskolin for 30 min and protein lysates were IP using either phospho-PKA substrate antibody or control IgG. Immunoprecipitates were resolved on SDS-PAGE and subjected to Western blotting with anti-pericentrin and anti-dynein antibodies. Whole cell lysates (*WCL*, 10 µg each) were used as input controls for Western immunoblots; gel lanes indicated by “X” are empty lanes, i.e., no protein loaded. Relative densitometry graph of Western immunoblots obtained from at least three independent experiments are presented (mean ± SEM, *p < 0.01; ***p < 0.001). Full-length blots are provided as Figure S16 in the Supplementary Material.

Protein kinase A has been known to interact with pericentrin in HEK293 cells (33) and phosphorylate *in vitro* expressed dynein intermediate chain one and light intermediate chain one subunits (34). This prompted us to investigate if these PCM proteins are substrates of PKA in human T-lymphocytes. For this purpose, T-cells were serum starved for 4 h followed by treatment with forskolin, a potent activator of adenylyl cyclase that elevates cAMP-dependent PKA activity, at a concentration of 30 µM for 30 min. Cellular lysates of untreated or forskolin pre-treated T-cells were pulled down using an antibody against the phosphorylated PKA consensus sequence RRXp(S/T) that detects proteins/peptides containing a phospho-Ser/Thr residue with Arginine at the -3 and -2 positions (35, 36) and subsequently Western immunoblotted for pericentrin and dynein. We detected that forskolin-induced activation of PKA increased the phosphorylated forms of both PCM proteins pericentrin and dynein (Figure 9A). An indirect interaction between CG-NAP and PKA substrates was also observed by immunoprecipitation assay using phospho-PKA substrate antibody (data not shown).

To determine the role of CG-NAP in facilitating the interaction between PKA and PCM proteins in motile T-cells, we knocked down CG-NAP in HuT78 T-cells using GapmeR technique and stimulated *via* LFA-1/ICAM-1. Cellular lysates from control or CG-NAP KD HuT78 cells were pulled down using phospho-PKA substrate antibody and subsequently Western immunoblotted for CG-NAP, pericentrin, and dynein. While there was no significant change in the interaction between dynein and PKA upon LFA-1 stimulation or CG-NAP KD in T-cells, significant reduction in the levels of pericentrin being pulled down by anti-PKARIIα antibody was detected in CG-NAP-depleted T-cells (Figure 9B). Loss of CG-NAP expression resulted in substantially decreased levels of forskolin-induced phosphorylation of both dynein and pericentrin (Figure 9C). These results suggest a critical involvement of CG-NAP in facilitating the phosphorylation of dynein and pericentrin by PKA, but the direct interaction between PKA and dynein is independent of CG-NAP or LFA-1 signal. The exact role of PKA-mediated phosphorylation of PCM proteins in T-cell motility deserves further investigation to advance our understanding of T-cell functioning. It might simply reflect that dynein and PKA will associate in a different steric configuration independent of CG-NAP.

## Discussion

**Figure 10:**
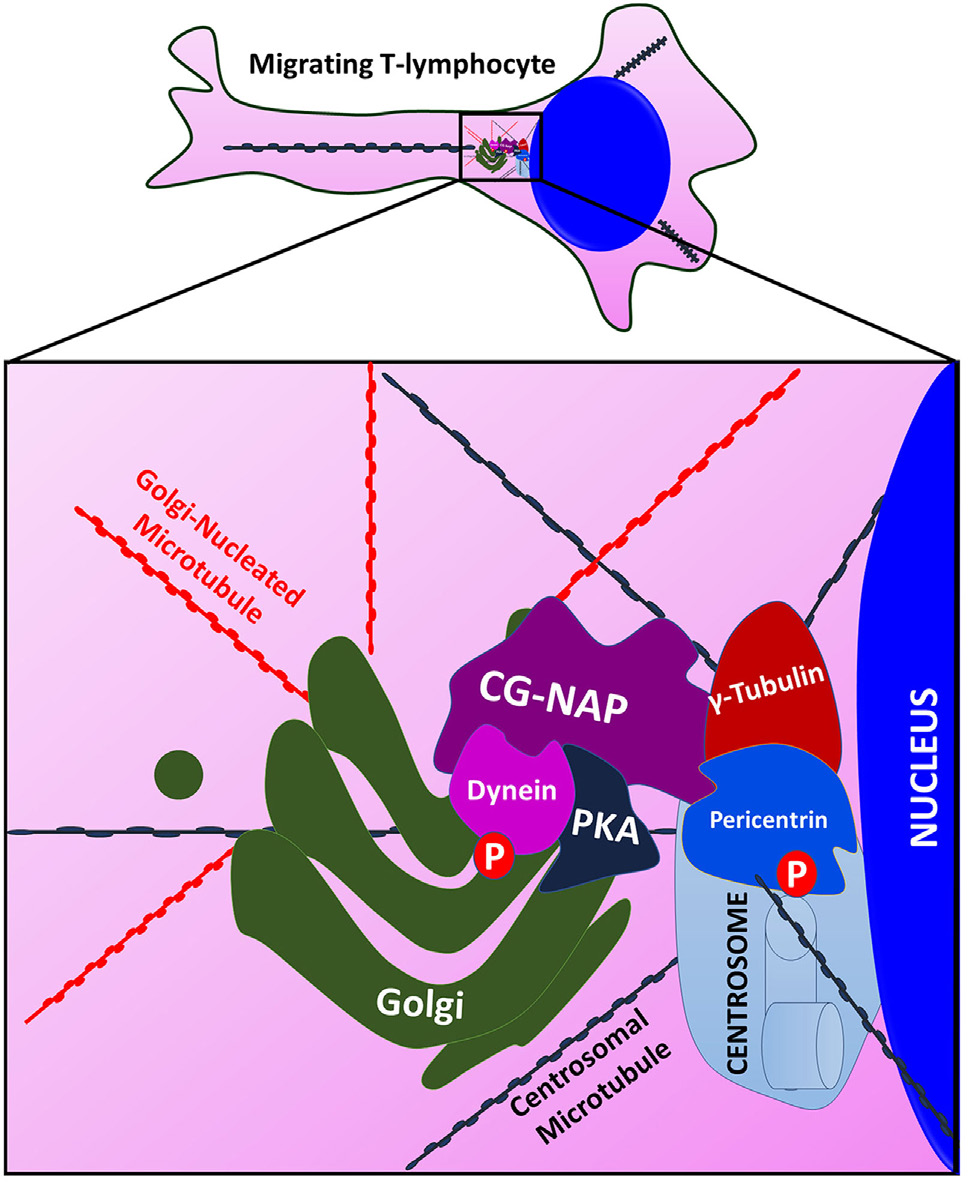
An illustration showing that Centrosome- and Golgi-localized protein kinase N-associated protein (CG-NAP) serves as a docking platform for protein kinase A (PKA) and pericentriolar material proteins to regulate microtubule nucleation in migrating T-lymphocytes. CG-NAP is localized to the centrosome where it interacts with pericentrin and γ-tubulin and Golgi. The giant adaptor protein also provides a docking platform for PKA for phosphorylation of its substrates dynein and pericentrin. These interactions aid in centrosomal and non-centrosomal microtubule nucleation in motile T-cells.

Maintenance of centrosomal architecture, microtubule nucleation, and anchoring of microtubule steric filaments are all crucial for cellular functions, including the highly active dynamic processes of T-cell motility. In this regard, current study provides strong evidence for the regulation of centrosomal and non-centrosomal microtubule nucleation in migrating T-cells by an adaptor protein CG-NAP. This giant 450 kDa adaptor protein is abundantly expressed in human primary T-cells and predominantly localized to the centrosomal regions, where it directly interacts with PKARIIα, pericentrin, and γ-tubulin in close proximity with the *cis*- and *trans*-Golgi structures. It also localizes in the vicinity of centrioles or outside the centrosome in the cytoplasm as “pericentriolar satellites” or “centriolar satellites.” The distinct confinement of CG-NAP at the microtubule nucleating organelles and pericentriolar satellites in human T-cells clearly indicate its crucial involvement in T-cell migration and other functions (Figure 10).

Previous study demonstrated that HuT78 T-cells transfected with a functional mutant form of CG-NAP were unable to display LFA-1-induced migratory phenotypes (4). Moreover, CG-NAP-depleted retinal pigment epithelial cell line RPE-1 showed defective cell migration (9). In this study, we observed that RNAi-mediated KD of CG-NAP in primary T-cells as well as T-cell line HuT78 caused significant inhibition of LFA-1/ICAM-1-induced T-cell migration. CG-NAP-depleted PBL T-cells displayed significantly reduced transwell chemotaxis toward the chemokine SDF-1α. However, another study showed that AKAP9 expression was dispensable for both chemotaxis and cell proliferation in T-cell-specific AKAP9 knockout mice (37). The discrepancy between our findings and the animal knockout studies could be attributed mainly to the different experimental conditions and migration model.

The organization and nucleation of microtubules at the centrosome require γ-tubulin, a protein that exists in a macromolecular complex called the γ-tubulin ring complex (γTuRC) (1, 9, 38, 39). Several pericentriolar proteins, including pericentrin/kendrin (10, 40), CDK5RAP2 (41, 42), myomegalin (43), ninein (41, 42), and CAP350 (44) have been implicated in the integration of the γTuRC with the centrosome. The centrosomal localization of PKA is accomplished through association of PKA holoenzyme with AKAPs, AKAP79 (2, 45), CG-NAP, and pericentrin (3, 5, 7, 33). In the microtubule regrowth assay, we detected that CG-NAP disassembled from the centrosome in both primary T-cells and HuT78 cells. Further, we demonstrated that PKARIIα and centrosomal proteins, γ-tubulin and pericentrin, were also displaced from the centrosome following microtubule depolymerization and that their localization became fragmented. Notably, γ-tubulin and pericentrin still remained associated with the disassembled CG-NAP complex under microtubule depolymerizing condition, suggesting that these associations are microtubule independent. In contrast, in HeLa cells, such protein-protein interactions were found to be microtubule-dependent (46). These data suggests the possibility of cell-type-specific nature of protein–protein interactions that might be unique to T-lymphocytes. Breakdown of pericentrin and γ-tubulin was only reported in muscle fibers following treatment with microtubule depolymerizing compound nocodazole (47). Silencing of CG-NAP profoundly disturbed centrosomal confinement of PKARIIα and also interrupted its interaction with pericentrin, which in turn perturbed microtubule nucleation in T-cells.

Centrosome- and Golgi-localized protein kinase N-associated protein was shown to be important for the structural maintenance of Golgi and for modulation of microtubule regrowth nucleation (9, 10, 15, 48). In agreement to these findings, KD of CG-NAP in HuT78 T-cells led to fragmentation of GM130 and TGN46. Nevertheless, super-resolution images of microtubule regrowth assay suggest that Golgi may not possibly be the site for non-centrosomal microtubule nucleation, which occurs in a CG-NAP-dependent manner. Noteworthy, microtubules can arise from other intracellular sites, such as cytoplasm, nuclear periphery, and kinetochores (49). Both pericentrin and dynein have previously been shown as PKA substrates in HEK-293 and other cell types (33, 34), and these proteins play important roles in microtubule nucleation (9, 47, 50, 51). PKA has been suggested to spatially regulate key signaling events that are critical for actin cytoskeletal remodeling and cell polymerization during migration in epithelial cells (52). PKA and PKA-mediated phosphorylation of multiple substrates are known to modulate TCR signaling and regulate immune functions at multiple levels, for example through NF-κB, MAPK, MAP/ERK kinase, Ras, Raf, phospholipase Cγ1/2, Csk, and RhoA (49, 53, 54). In addition, a growing literature suggests an involvement of PKA in actin cytoskeletal dynamics, cell adhesion, and migration. In this context, our findings highlight a critical role of CG-NAP in LFA-1/ICAM-1-mediated T-cell migration and chemotaxis potentially mediated in part through interactions between PKA and its substrates. In support to our findings, an earlier study showed that suppression of AKAP450 expression impaired signaling events at the immunological synapse, including phosphorylation of phospholipase Cγ and protein kinase C-θ (55).

In conclusion, AKAP450/CG-NAP plays a central role as a downstream effector of LFA-1 signaling by (i) regulating microtubule nucleation, (ii) maintaining centrosomal and Golgi architecture, and (iii) recruiting and coordinating multi-protein complexes, which are essential for the active and highly dynamic process of T-cell motility.

## Author Contributions

DK and NV conceived the experiments. STO, MLC, MF, PP, and AK designed and performed the experiments. GW performed 3D-SIM microscopy. DK and NV analyzed processed data. NV, MC, and STO wrote the manuscript with critical revisions by the other authors. All authors approved the final manuscript version.

## Conflict of Interest Statement

The authors declare that the research was conducted in the absence of any commercial or financial relationships that could be construed as a potential conflict of interest.
