# Supplementary material for: Centrosome- and Golgi-Localized Protein Kinase N-Associated Protein Serves As a Docking Platform for Protein Kinase A Signaling and Microtubule Nucleation in Migrating T-Cells
Source: Front Immunol. 2018 Mar 1;9:397. doi: 10.3389/fimmu.2018.00397 (PMC5837996; doi:10.3389/fimmu.2018.00397)
Supplement: Supplementary file 1 [file Presentation_1.PDF]

Supplementary information

CG-NAP serves as a docking platform for PKA signaling and microtubule nucleation in migrating T-cells

Seow Theng Ong[1], Madhavi Latha Somaraju Chalasani[1], M. H. U. T. Fazil[1], Praseetha Prasannan[1], Atish Kizhakeyil[1], Graham D. Wright[2], Dermot Kelleher[1,3,4], Navin Kumar Verma[1,5]

[1]Lee Kong Chian School of Medicine, Nanyang Technological University, Singapore
[2]Institute of Medical Biology, A*STAR, Singapore
[3]Department of Medicine, University of British Columbia, Vancouver, BC, Canada
[4]Department of Biochemistry and Molecular Biology, University of British Columbia, Vancouver, BC, Canada
[5]Singapore Eye Research Institute, Singapore

SUPPLEMENTARY FIGURES S1 - S16

SUPPLEMENTARY VIDEO 1: 3D-SIM projection showing co-localization of CG-NAP (*green*) and γ-tubulin (*red*) in human primary T-lymphocytes.

SUPPLEMENTARY VIDEO 2: 3D-SIM projection showing co-localization of CG-NAP (*green*) and GM130 (*red*) in human primary T-lymphocytes.

SUPPLEMENTARY VIDEO 3: A volumetric view of 3D-SIM image stack of HuT78 T-cells co-stained for CG-NAP (*red*), α-tubulin (*green*) and PKARIIα (*cyan*).

SUPPLEMENTARY VIDEO 4: 3D projection of super-resolution microscopy image showing that CG-NAP (*red*) anchors pericentrin (*magenta*) at multiple microtubule nucleation sites (*green*) in control HuT78 T-cells. Still images are shown in Figure S5A in Supplementary Material.

SUPPLEMENTARY VIDEO 5: 3D projection of super-resolution microscopy image showing pericentrin (*magenta*) and microtubule nucleation (*green*) in CG-NAP (*red*) knockdown HuT78 T-cells. Still images are shown in Figure S5C in Supplementary Material.

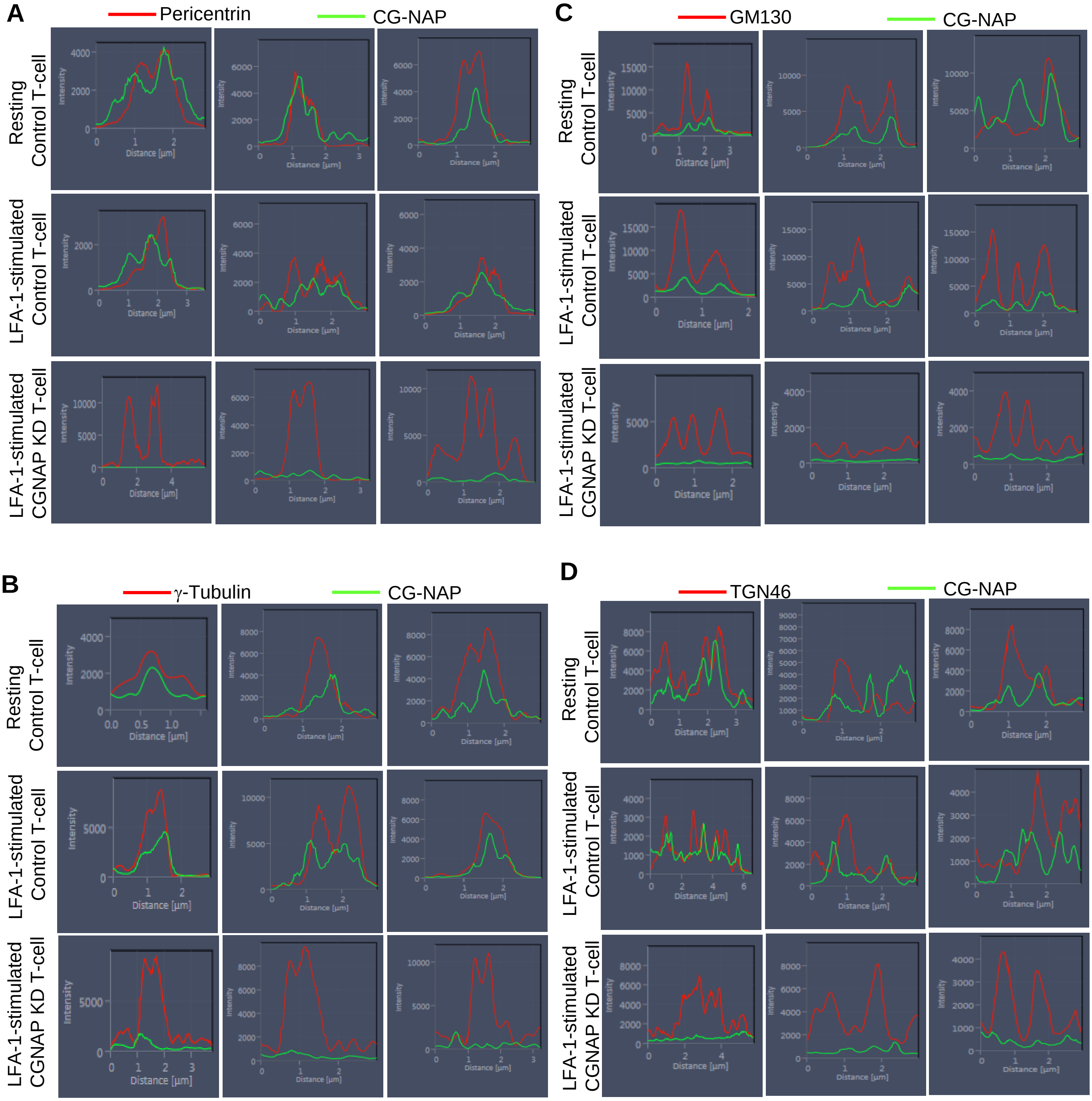

SUPPLEMENTARY FIGURE S1. Fluorescence signal intensity profiles of CG-NAP with PCM and Golgi proteins in confocal microscopic images obtained from three different sets of experiments. Line scan graphs show the overlap fluorescence intensity of CGNAP (*green*) with pericentrin (*red*, **A**), γ-tubulin (*red*, **B**), GM130 (*red*, **C**), or TGN46 (*red*, **D**).

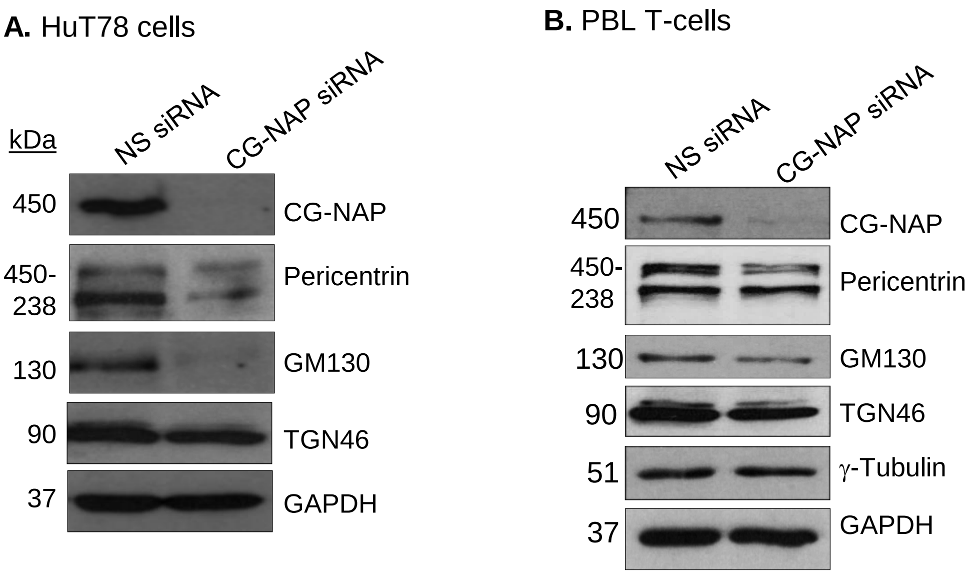

SUPPLEMENTARY FIGURE S2. Western blots showing the effect of siRNA-mediated knockdown of CG-NAP on the expression of PCM and Golgi proteins. HuT78 T-cells (**A**) and primary PBL T-cells (**B**) were nucleofected with non-specific (*NS*) or CG-NAP targeting siRNA (100 nM each) for 48 h and lysed. Cellular lysates were analyzed for the expression of CG-NAP, pericentrin, GM130, TGN46 and γ-tubulin. GAPDH was used as a loading control. Experiments were repeated 3 times and representative blots are shown.

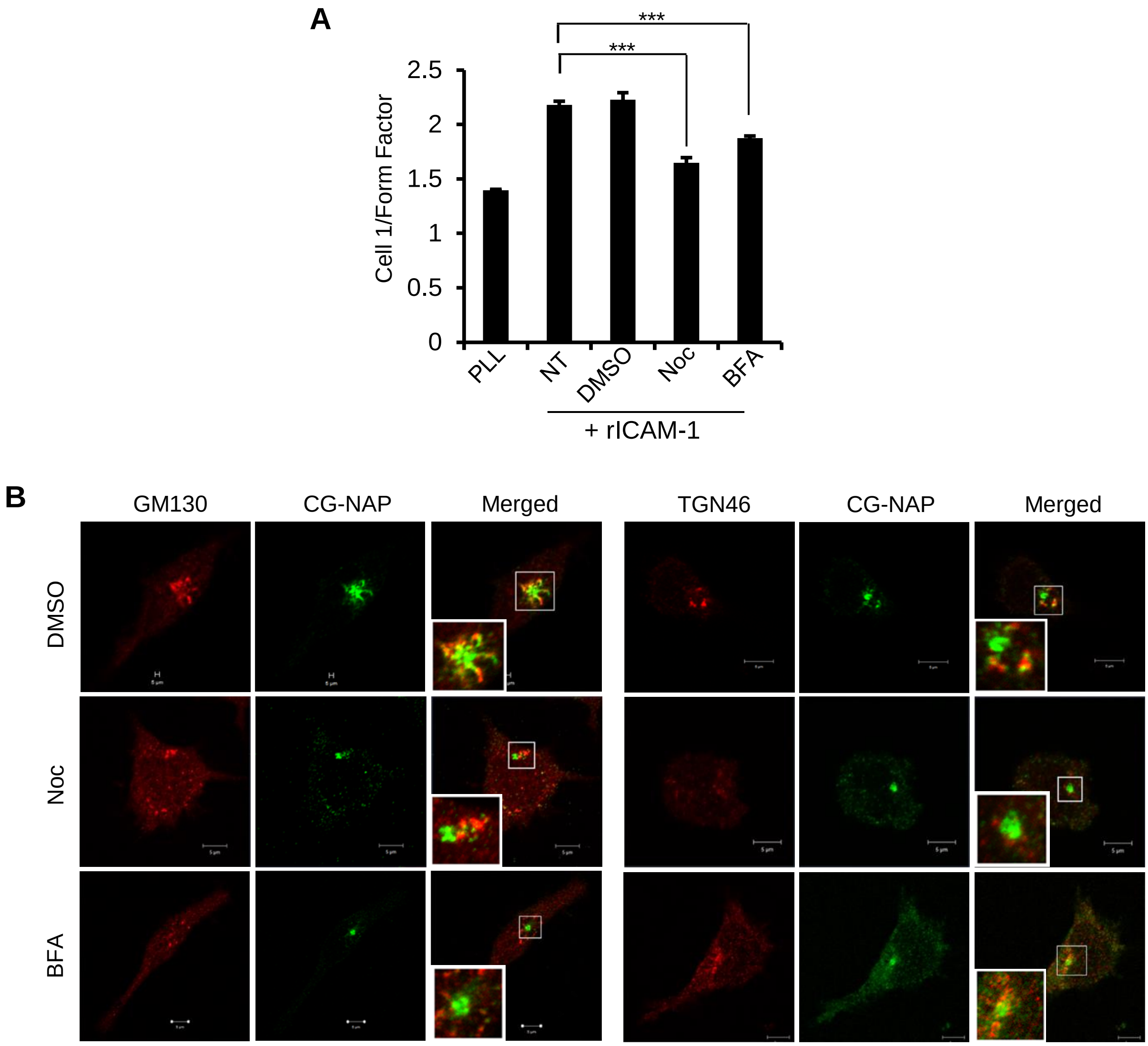

SUPPLEMENTARY FIGURE S3. Treatment with nocodazole or brefeldin-A causes Golgi fragmentation in T-cells**.** (**A**) Primary T-cells were pretreated with either DMSO control or 10 µM nocodazole (*Noc*) or 10 µM brefeldin A (*BFA*) and seeded on rICAM-1-coated plate for migration. T-cell migratory phenotypes were quantified by High Content Analysis. Cell 1/form factor (mean±S.E.M.) values were obtained from an automated cell-by-cell analysis of >2000 cells/well in 96-well plates, treated in triplicate wells in 3 independent experiments. ***p<0.001 (**B**) HuT78 T-cells pretreated in a similar way were immunostained with antibodies against CG-NAP (*green*) and GM130 or TGN46 (*red*) and visualized by confocal microscopy.

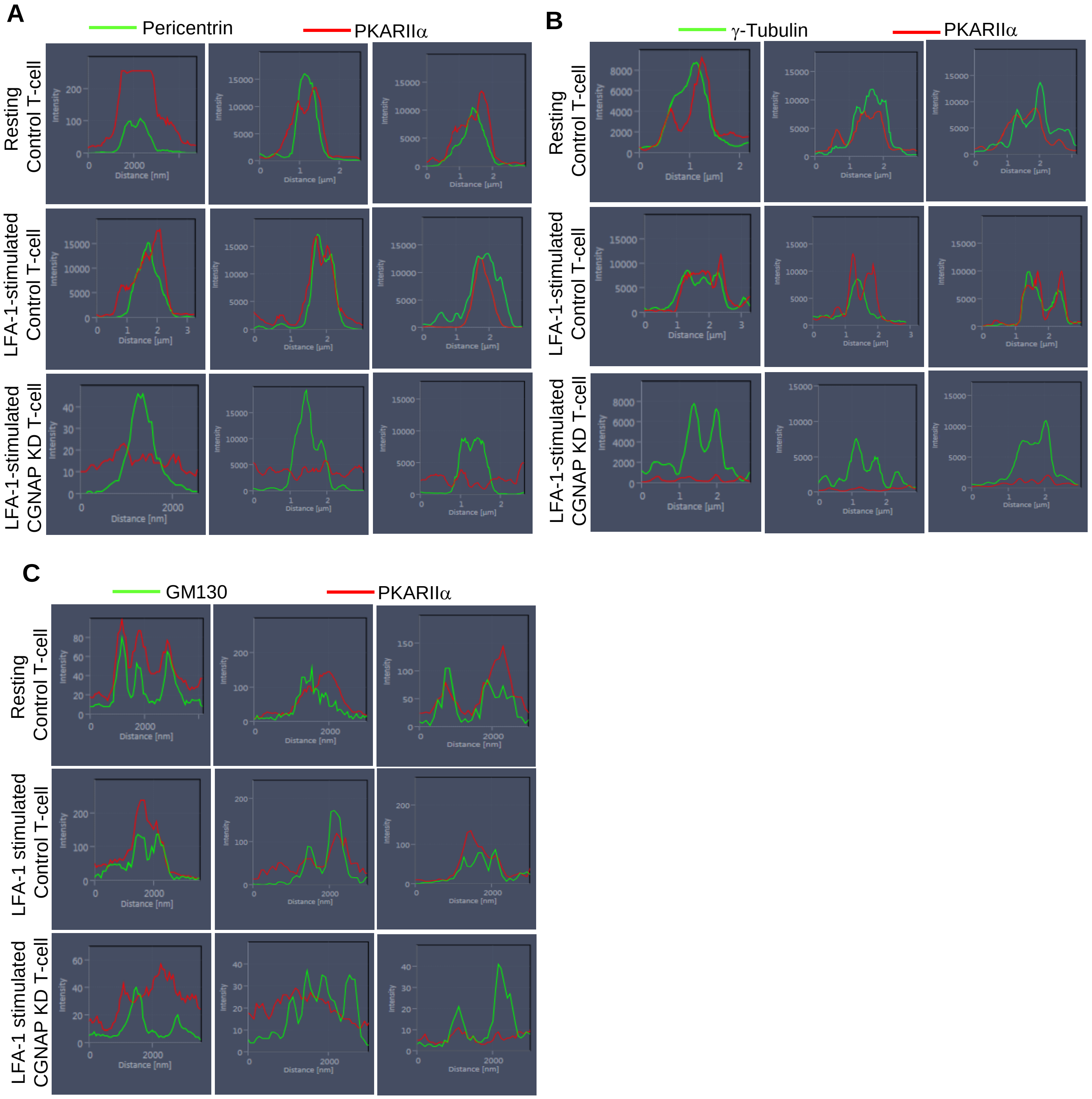

SUPPLEMENTARY FIGURE S4. Fluorescence signal intensity profiles of PKARIIα with pericentrin, γ-tubulin and GM130 for the confocal microscopic images obtained from three different sets of experiments. Line scan graphs show the overlap fluorescence intensity of PKARIIα (*red*) with pericentrin (*green*, **A**), γ-tubulin (*green*, **B**), or GM130 (*green*, **C**).

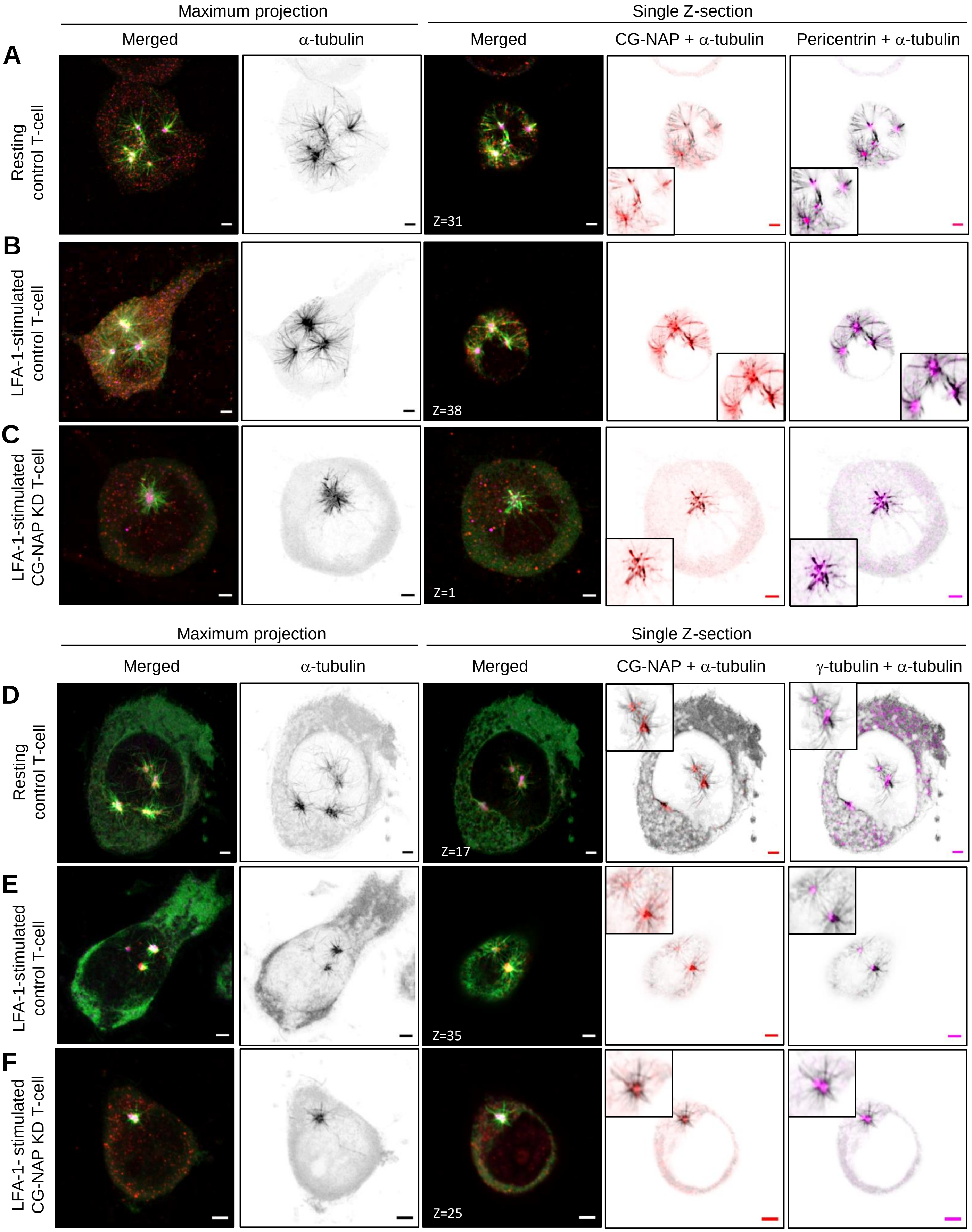

SUPPLEMENTARY FIGURE S5. CG-NAP regulates centrosomal microtubule nucleation in motile T-cells. Control and CG-NAP knockdown (*KD*) HuT78 T-cells were either unstimulated (*resting*) or stimulated *via* LFA-1/ICAM-1 for 2 h, subjected to microtubule regrowth assay for 20 sec and fixed. Cells were immunostained for CG-NAP (*red*), α-tubulin (*green*) and pericentrin (*magenta*) (**A-C**) or γ-tubulin (*magenta*) (**D-F**) and analyzed by confocal microscopy. Microtubule images were inverted into negative grey-scale and selected regions were magnified as insets for better visualization. Images show (**A, D**) control unstimulated, (**B, E**) LFA-1-stimulated and (**C, F**) CG-NAP depleted T-cell. Scale bar: 2 μm.

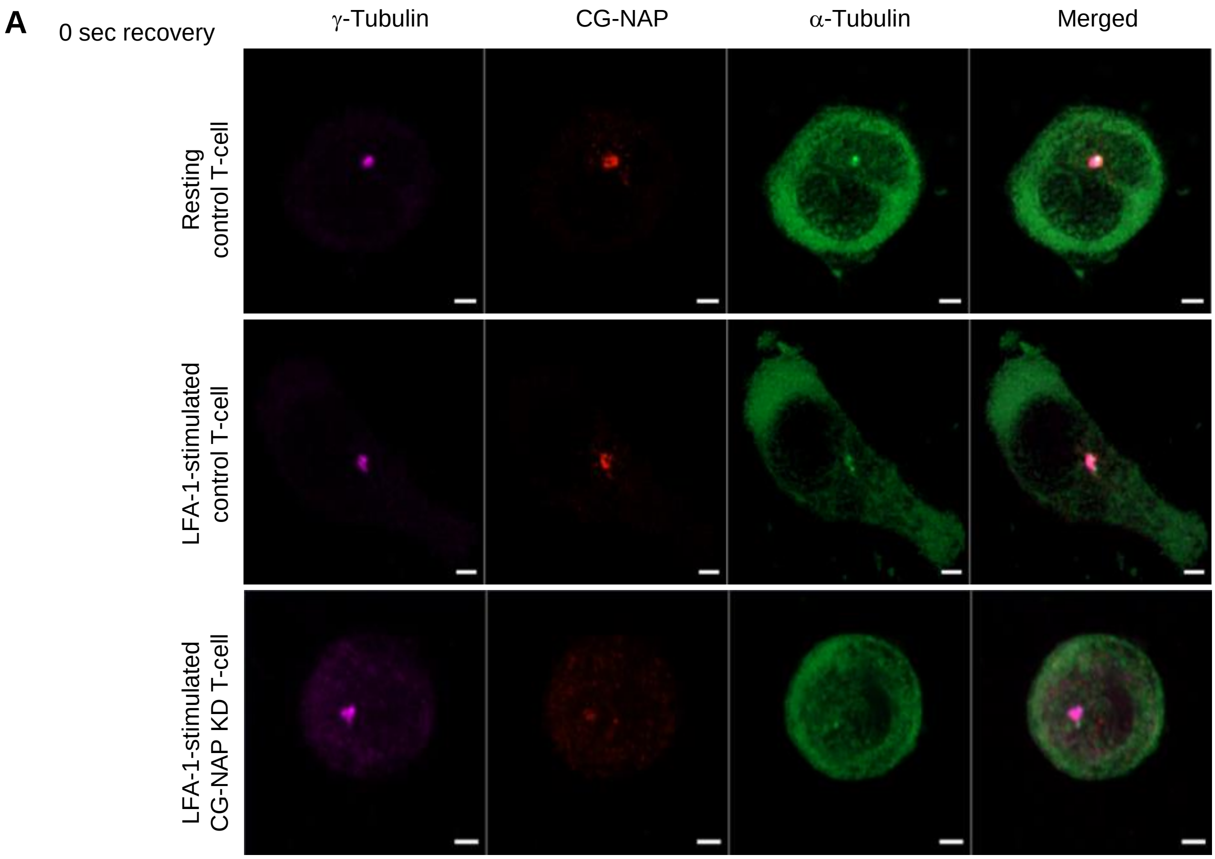
A
0 sec recovery
γ-Tubulin
CG-NAP
α-Tubulin
Merged
Resting control T-cell
LFA-1-stimulated control T-cell
LFA-1-stimulated CG-NAP KD T-cell

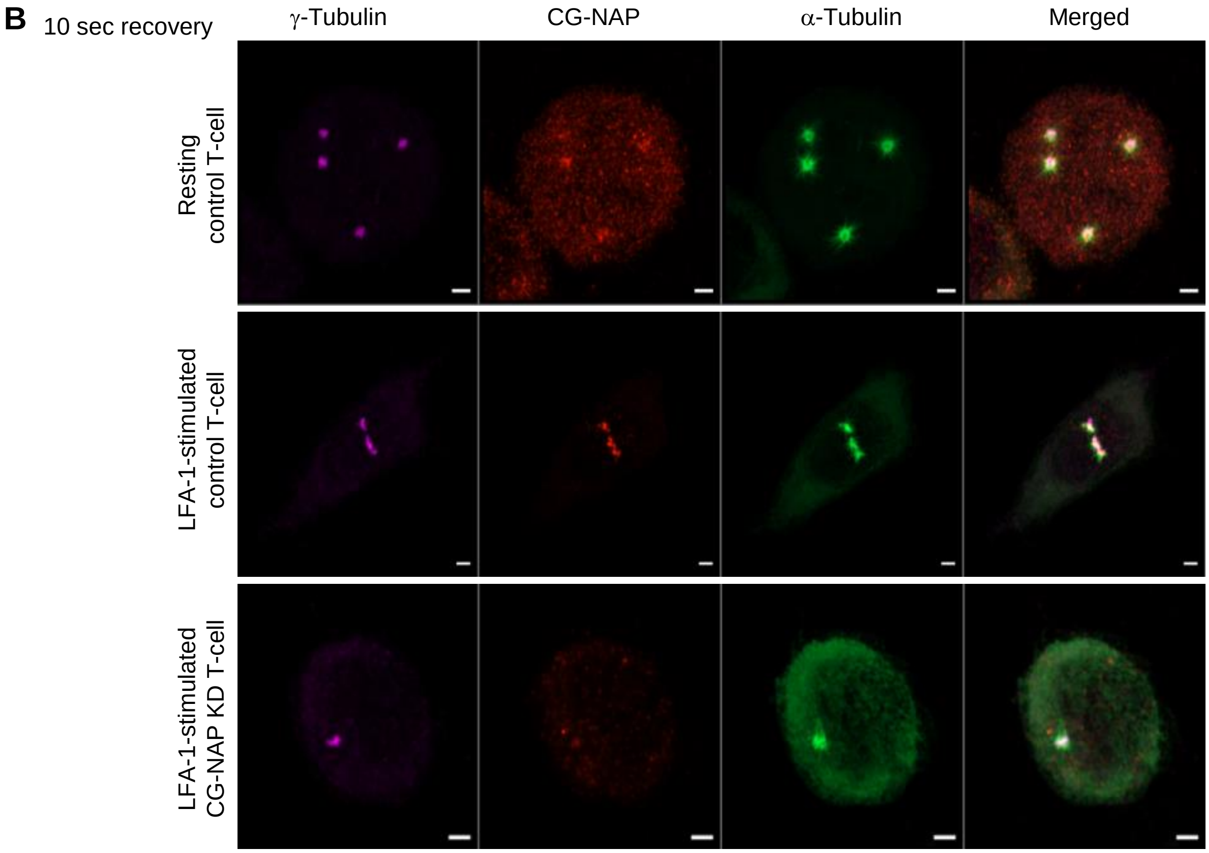
B
10 sec recovery
γ-Tubulin
CG-NAP
α-Tubulin
Merged
Resting control T-cell
LFA-1-stimulated control T-cell
LFA-1-stimulated CG-NAP KD T-cell

To continue…

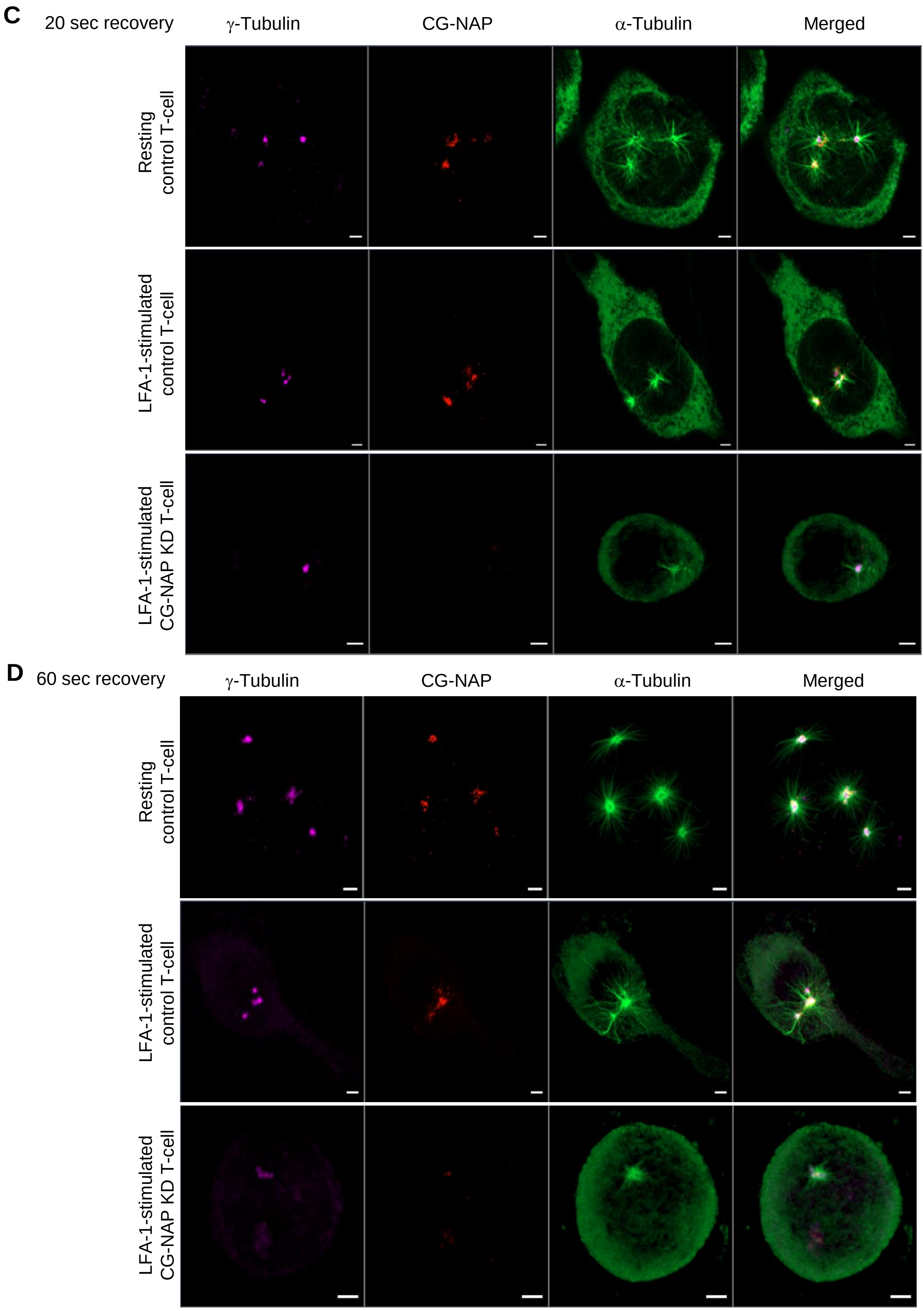

SUPPLEMENTARY FIGURE S6. CG-NAP regulates centrosomal microtubule nucleation (γ-tubulin) in HuT78 T-cells. Control and CG-NAP knockdown (*KD*) HuT78 T-cells were either un-stimulated (*resting*) or stimulated *via* LFA-1/ICAM-1 for 2 h, subjected to microtubule regrowth assay for 0 sec (**A**), 10 sec (**B**), 20 sec (**C**) or 60 sec (**D**) and fixed. Cells were co-stained for γ-tubulin (*magenta*), CG-NAP (*red*) and α-tubulin (*green*) and imaged by confocal microscopy. Scale bar: 2 µm.

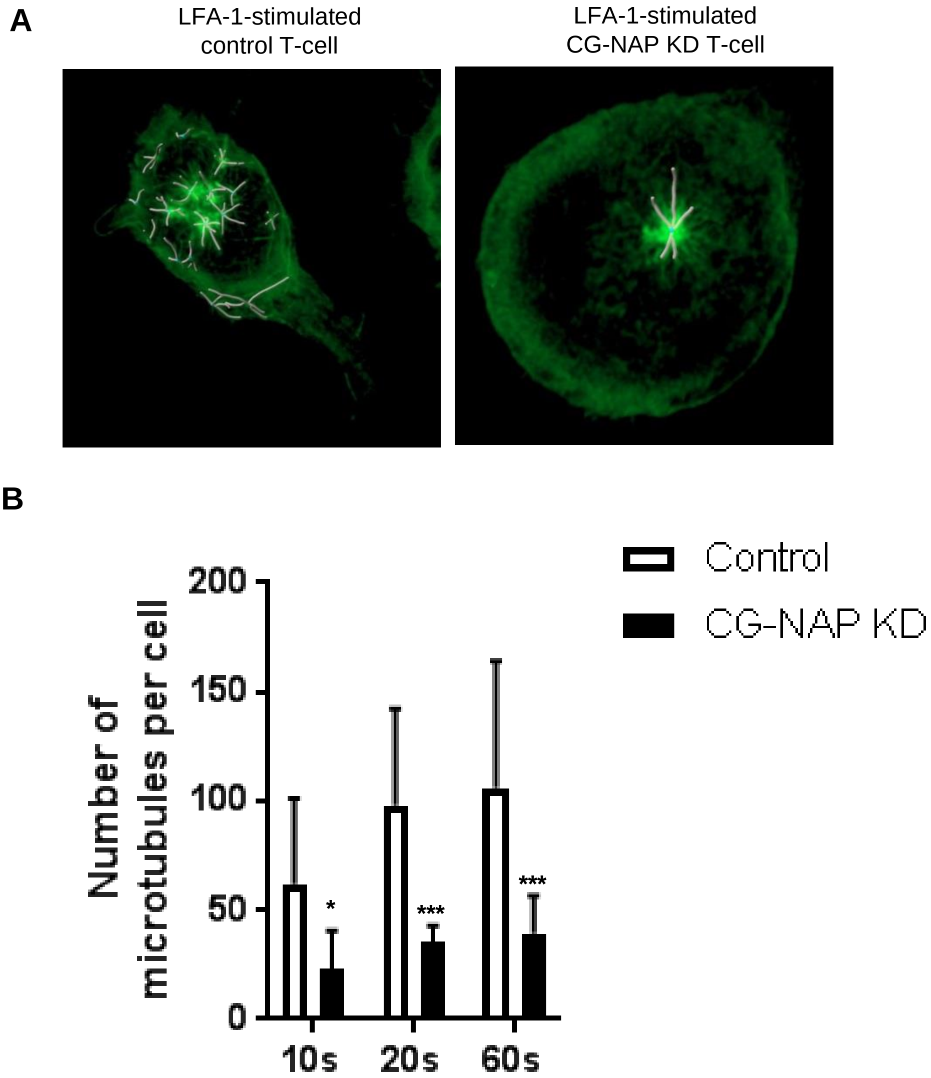

SUPPLEMENTARY FIGURE S7. Effect of CG-NAP knockdown on the ability of LFA-1-stimulated T-cell microtubules to regrow. Control and CG-NAP knockdown (*KD*) HuT78 cells were stimulated *via* LFA-1/ICAM-1 for 2 h, subjected to microtubule depolymerization and regrowth for 10 sec, 20 sec or 60 sec and fixed. (**A**) Cells were immunostained for α-tubulin (*green*) and imaged by confocal microscopy, 10 sec. (**B**) The number of microtubules per cell in LFA-1-stimulated cells were quantified by Imaris software and presented. Mean ±S.E.M., n=10 from at least 3 independent experiments, *p<0.05, ***p<0.001.

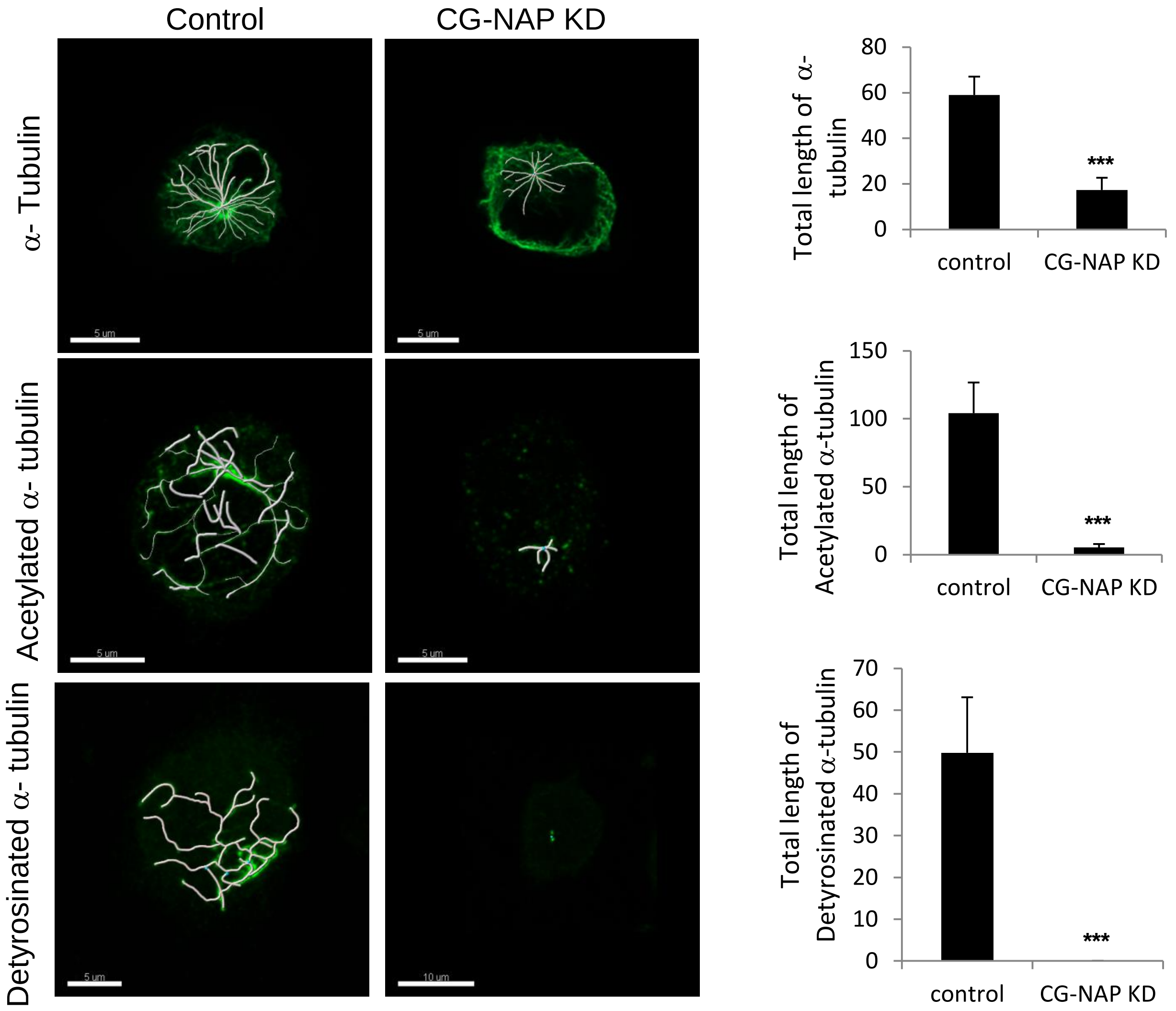

SUPPLEMENTARY FIGURE S8. Effect of CG-NAP knockdown on α-tubulin post-translational modifications. Control and CG-NAP knockdown (*KD*) HuT78 cells were immunostained with antibodies for α-tubulin, acetylated-α-tubulin or detyrosinated-α-tubulin and analysed by confocal microscopy. Imaris software was used to perform 3D projection of the confocal z-stacks and microtubule lengths were analysed using filament tracing tool. At least 10 images from 3 different experiments were analysed and results were pooled and presented as graphs (means ± S.E.M). ***p<0.001.

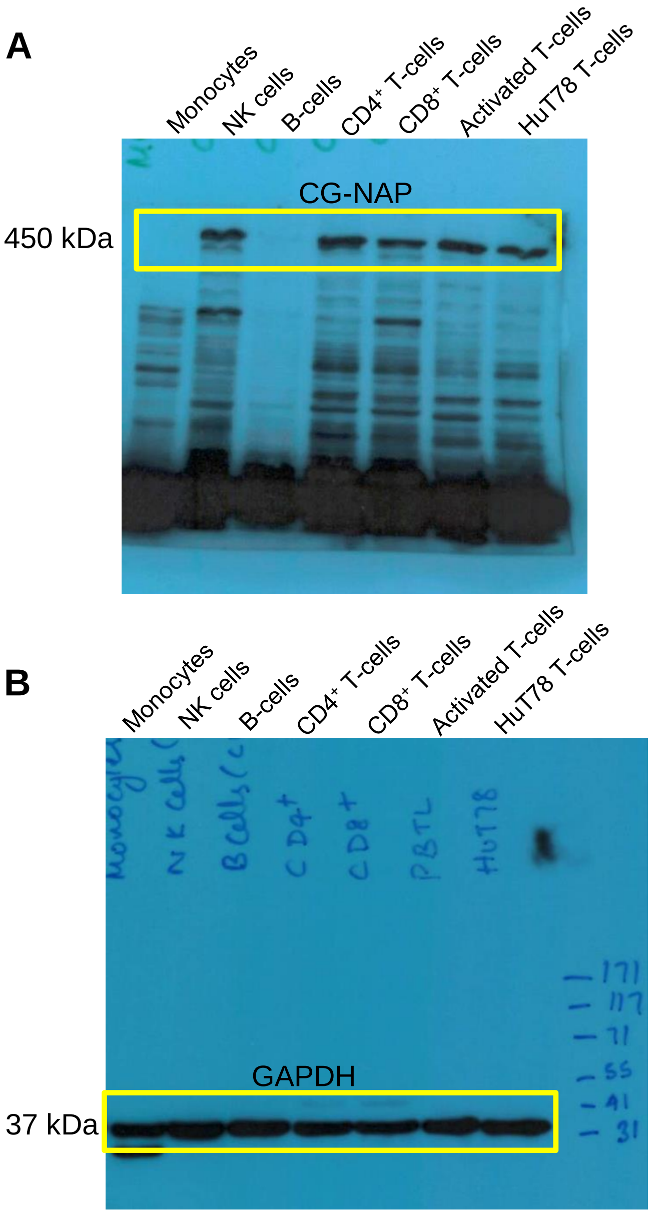

SUPPLEMENTARY FIGURE S9. Western immunoblot data showing the relative expression of CG-NAP protein in various immune cell subtypes. Cellular lysates (10 µg each) obtained from monocytes, NK-cells, B-cells, CD4$^+$ T-cells, CD8$^+$ T-cells, activated PBL T-cells and HuT78 T-cell line were analyzed by Western immunoblotting (**A**). Blots were re-probed with GAPDH as a loading control (**B**). The box indicates the cropped portions that were used in Figure 1A of the main article.

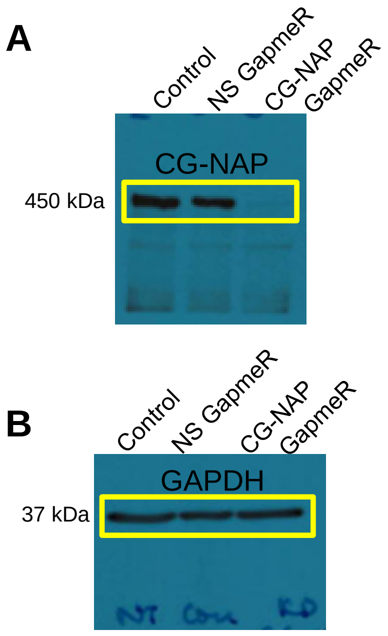

SUPPLEMENTARY FIGURE S10. Western immunoblot data showing GapmeR-mediated gene silencing of CG-NAP protein in human T-cells. HuT78 cells (untreated, *control*) were treated with 500 nM non-specific GapmeR (*NS GapmeR*) or GapmeR targeted against CG-NAP (*CG-NAP GapmeR*) for 48 h and analysed by Western immunoblotting (**A**). Blots were re-probed with GAPDH as a loading control (**B**). The box indicates the cropped portions that were used in Figure 2A of the main article.

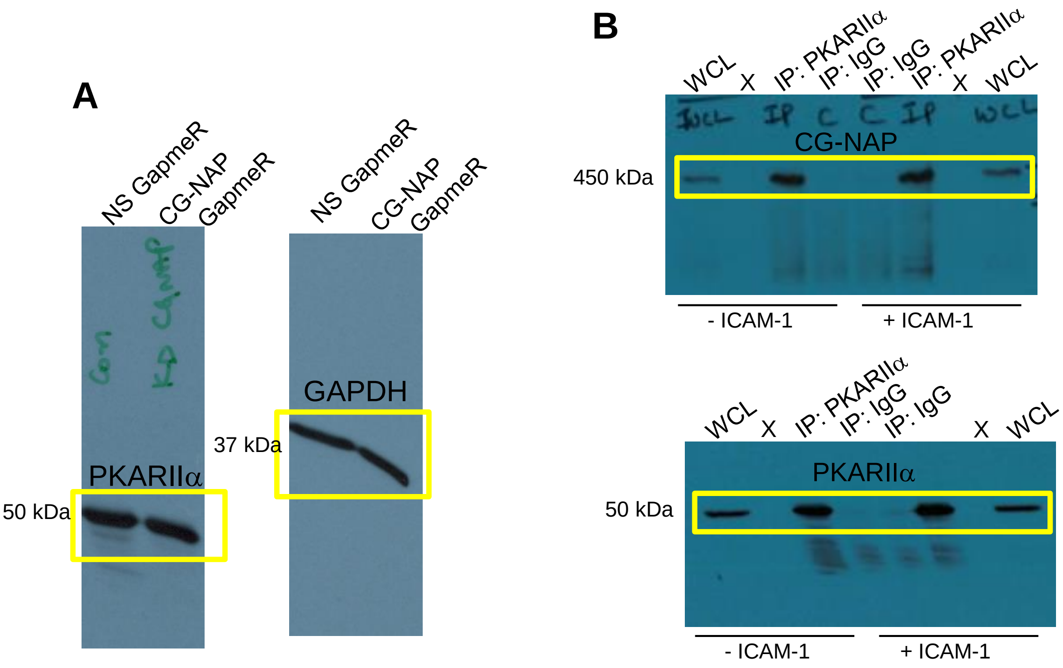

SUPPLEMENTARY FIGURE S11. Western immunoblot data showing an interaction between CG-NAP and PKARIIα proteins in human T-cells. (**A**) HuT78 cells were treated with 500 nM non-specific GapmeR (*NS GapmeR*) or GapmeR targeted against CG-NAP (*CG-NAP GapmeR*) for 48 h and analysed for their effect on the expression of PKARIIα by Western immunoblotting. GAPDH used as a loading control. (**B**) Cellular lysates from unstimulated or LFA-1/ICAM-1-stimulated HuT78 cells were immunoprecipitated (*IP*) using either anti-PKARIIα antibody or control IgG. Immunoprecipitates were resolved on SDS-PAGE and subjected to Western immunoblotting with anti-CG-NAP and anti-PKARIIα antibodies. The box indicates the cropped portions that were used in Figures 3C and 3D of the main article.

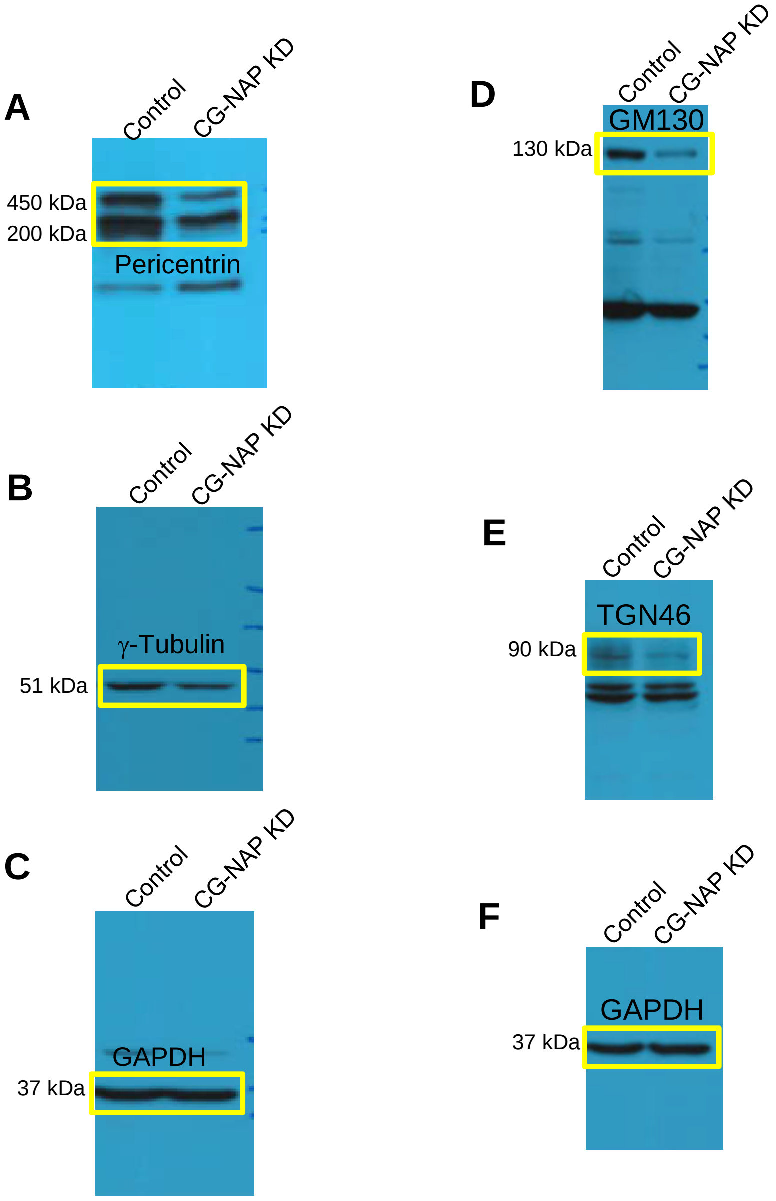

SUPPLEMENTARY FIGURE S12. Western immunoblot data showing decrease in the expression levels of centrosomal proteins due to CG-NAP knockdown in human T-cells. Cellular lysates from control and CG-NAP knock-down (*CG-NAP KD*) HuT78 cells (10 µg each) were analysed for the expression levels of pericentrin (**A**), γ-tubulin (**B**), GM130 (**D**) and TGN46 (**E**) by Western immunoblotting. GAPDH used as a loading control (**C, F**). The box indicates the cropped portions that were used in Figure 4C and 4F of the main article.

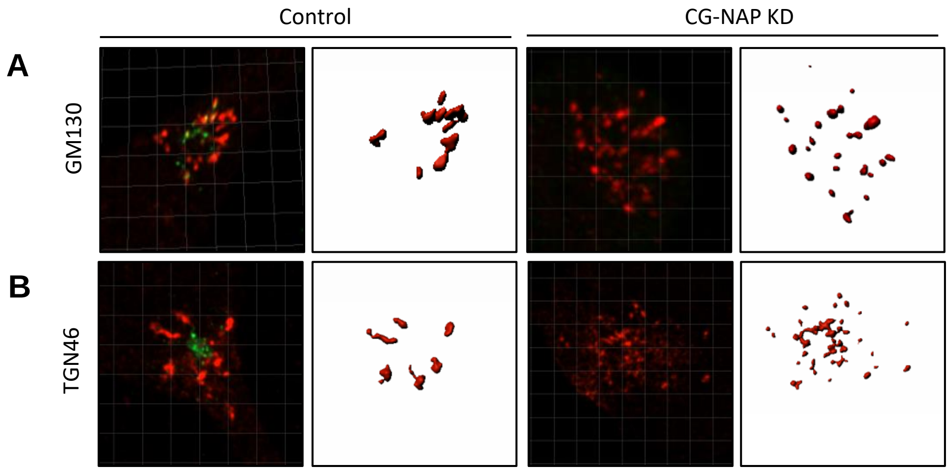

SUPPLEMENTARY FIGURE S13. Image quantitation of Golgi fragmentation. Control and CG-NAP knockdown (*ND*) HuT78 cells were stained with CG-NAP (*green*) and GM130 (**A**) or TGN46 (**B**) (*red*). Based on the Golgi staining, Imaris software was used to perform object rendering and the object surface volume was automatically quantified. Golgi structures with object volume < 0.2 μm^3 were considered fragmented. The percentage of cells with Golgi fragmentation is presented in Figure 4G. At least 10 images from 3 independent experiments were analysed and representative sets of images are shown.

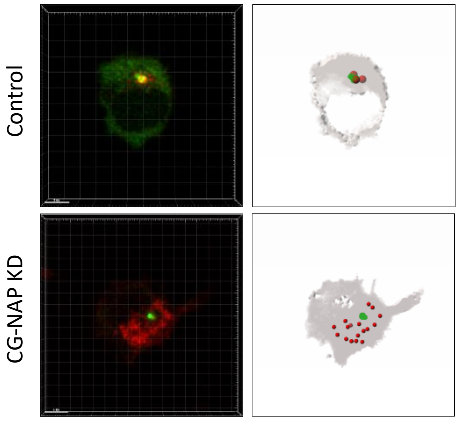

SUPPLEMENTARY FIGURE S14. Image analysis for centrosomal localisation of PKARIIα. Control and CG-NAP knockdown (*KD*) HuT78 cells were stained with centrosomal marker γ-tubulin (*green*) and PKARIIα (*red*). Imaris cell module was used to quantify the distance of PKARIIα structures to centrosome. PKARIIα structures with < 0.2 μm distance to centrosome were considered as confined to the centrosomal region. The percentage of cells with loss of centrosomal localization of PKARIIα is presented in Figure 5D. At least 10 images from 3 independent experiments were analysed and representative sets of images are shown.

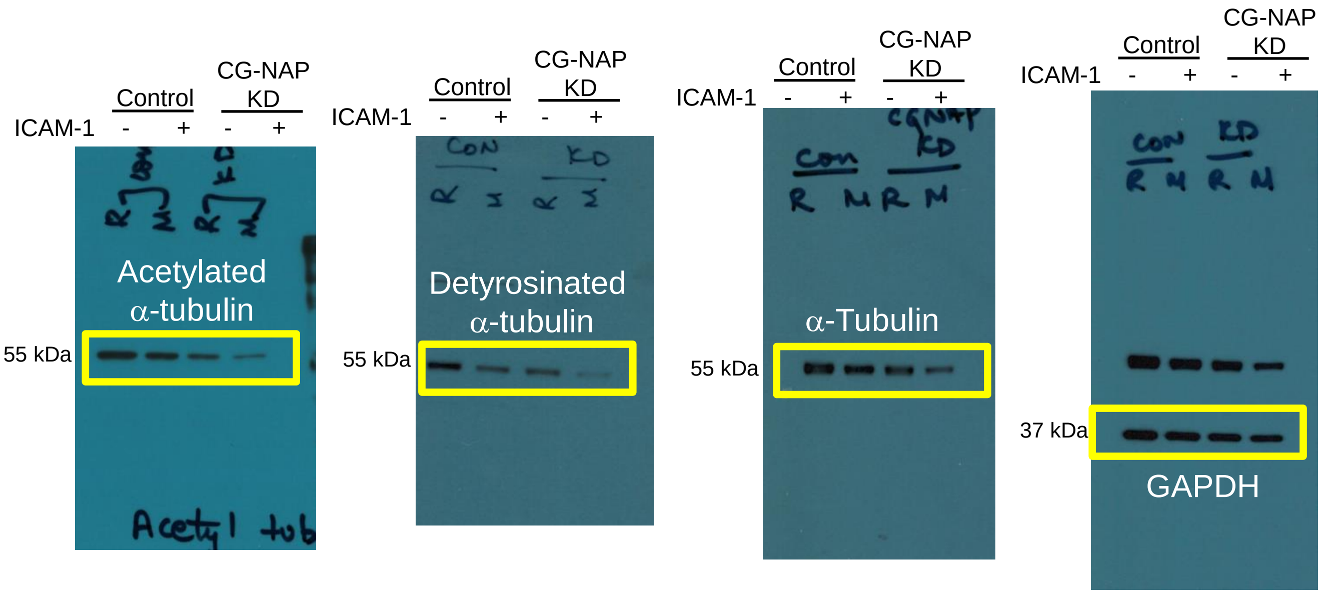

SUPPLEMENTARY FIGURE S15. Western immunoblot data showing the effect of CG-NAP knockdown on the post-translational modifications of α-tubulin in human T-cells. Cellular lysates from control and CG-NAP knockdown (*CG-NAP KD*) HuT78 cells (10 µg each) were analysed for acetylated and detyrosinated forms of α-tubulin. Blots were re-probed with GAPDH as a loading control. The box indicates the cropped portions that were used in Figure 8D of the main article.

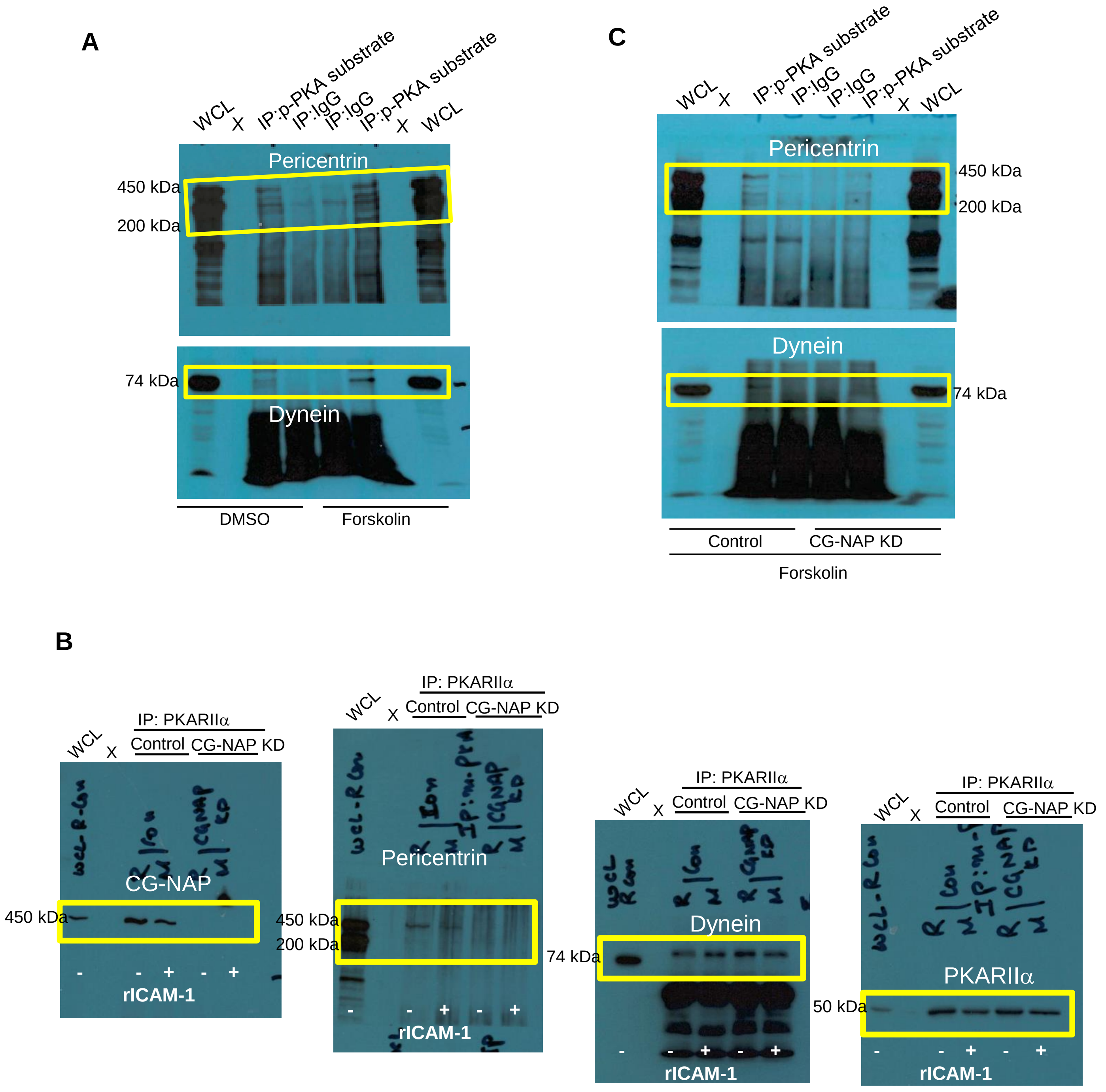

SUPPLEMENTARY FIGURE S16. Immunoblot analysis of PKA substrates in T-cells. (**A**) HuT78 cells were serum starved for 4 h and then treated with DMSO (control) or 30 µM forskolin for 30 min and lysed. Protein lysates were immunoprecipitated (*IP*) using either phospho-PKA substrate antibody (p-PKA substrate) or control IgG. Immunoprecipitates were resolved on SDS-PAGE and subjected to Western blotting with pericentrin and dynein antibodies. (**B**) Unstimulated or LFA-1-stimulated control or CG-NAP KD HuT78 cells were lysed, immunoprecipitated using anti-PKARIIα antibody and subjected to Western blotting with anti-CG-NAP, pericentrin, dynein and PKARIIα antibodies. (**C**) Control or CG-NAP knockdown (*KD*) HuT78 cells were treated with 30 µM forskolin for 30 min and protein lysates were immunoprecipitated using either phospho-PKA substrate antibody or control IgG. Immunoprecipitates were resolved on SDS-PAGE and subjected to Western blotting with anti-pericentrin and anti-dynein antibodies. Whole cell lysates (WCL, 10 µg each) were used as input controls for Western immunoblots; gel lanes indicated by “X” are empty lanes, *i.e*. no protein loaded. The box indicates the cropped portions that were used in Figure 9 of the main article.
